# The New PI3K/mTOR Inhibitor GNE-477 Inhibits the Malignant Behavior of Human Glioblastoma Cells

**DOI:** 10.3389/fphar.2021.659511

**Published:** 2021-07-26

**Authors:** Yixuan Wang, Heng Shen, Qian Sun, Linyao Zhao, Hao Liu, Liguo Ye, Yang Xu, Jiayang Cai, Yuntao Li, Lun Gao, Yinqiu Tan, Baohui Liu, Qianxue Chen

**Affiliations:** ^1^ Department of Neurosurgery, Renmin Hospital of Wuhan University, Wuhan, China; ^2^ Central Laboratory, Renmin Hospital of Wuhan University, Wuhan, China; ^3^ Department of Neurosurgery, Suizhou Hospital, Hubei University of Medicine, Suizhou, China

**Keywords:** GNE-477, GBM, PI3K/mTOR signaling pathway, proliferation, SC79

## Abstract

The most common primary central nervous system tumor in adults is glioblastoma multiforme (GBM). The high invasiveness of GBM cells is an important factor leading to inevitable tumor recurrence and a poor prognosis of patients. GNE-477, a novel PI3K/mTOR inhibitor, has been reported to exert antiproliferative effects on other cancer cells. However, researchers have not clearly determined whether GNE-477 produces antitumor effects on GBM. In the present study, GNE-477 significantly inhibited the proliferation, migration and invasion of U87 and U251 cells. In addition, GNE-477 also induced apoptosis of GBM cells, arresting the cell cycle in G0/G1 phase. More importantly, GNE-477 also reduced the levels of AKT and mTOR phosphorylation in the AKT/mTOR signaling pathway in a concentration-dependent manner. An increase in AKT activity induced by SC79 rescued the GNE-477-mediated inhibition of GBM cell proliferation and apoptosis. The antitumor effects of GNE-477 and the regulatory effects on related molecules were further confirmed *in vivo* using a nude mouse intracranial xenograft model. In conclusion, our study indicated that GNE-477 exerted significant antitumor effects on GBM cells *in vitro* and *in vivo* by downregulating the AKT/mTOR pathway.

## Introduction

Gliomas account for approximately 80% of all brain-related malignancies (Ostrom et al., 2015), have an incidence of 5.26 per 100,000 in the United States (Omuro and Deangelis, 2013), account for approximately 2.7% of all cancer-related deaths, and are expected to account for more than 23,000 new cases annually (Omuro and Deangelis, 2013). However, among them, glioblastoma is the most common glioma (accounting for approximately 45% of all gliomas), and combined with the characteristics of tumor heterogeneity, glioblastoma is difficult to successfully cure (Ostrom et al., 2014). Current multimodal therapies for glioblastoma combining surgery, radiotherapy, systemic therapy (chemotherapy or targeted therapy) and supportive therapy have achieved some progress, but the overall prognosis is still poor, the long-term survival rate is very low, and the short-term survival rate has improved over time. However, the 5-years survival rate remains relatively constant, and the 5-years survival rate after diagnosis remains relatively constant at only 5.8% (Tan et al., 2020). Among these factors, the most distressing is that at least 50% of patients with GBM do not respond to TMZ, the most effective treatment (Lee, 2016). Therefore, an urgent need is to develop new, highly effective drugs and innovative therapies to improve patient prognosis and quality of life.

In terms of its importance in cancer, the PI3K signaling cascade is more suitable to be called a highway than a pathway. The phosphoinositide three kinase (PI3K)/AKT/mammalian rapamycin target (mTOR) axis is frequently altered in various human cancers, thereby promoting tumor growth, proliferation and survival (Courtney et al., 2010; Dienstmann et al., 2014). Complementarily, the lack of dominant single oncogenic “driver” mutations and diversified activation of signaling pathways in cancer cells due to intratumor heterogeneity has become increasingly clear, and strategies targeting only a single receptor or signaling pathway have become less likely to succeed against GBM (Sathornsumetee et al., 2007). Therefore, the idea of designing small-molecule compounds that simultaneously target multiple kinases to achieve inhibition of multiple pathways has attracted increasing interest. GNE-477, a novel dual inhibitor of PI3K/mTOR, has been demonstrated to inhibit the growth of renal cell carcinoma cells *in vitro* and *in vivo* (Ye et al., 2020). PI3K/mTOR is also a cascade reaction of the main pathway in GBM, but researchers have not determined whether GNE-477 exerts a significant antitumor effect on GBM.

Therefore, in this study, we provide evidence that GNE-477 inhibits the proliferation of glioblastoma cells, induces cell cycle arrest in G1 phase, and delays the invasion and migration of U87 and U251 cells. At the same time, it also promotes apoptosis in a dose-dependent manner. These effects are mediated by inhibition of the Akt/mTOR signaling axis, as more clearly evidenced by SC79, an AKT activator. More surprisingly, as shown in subsequent studies, GNE-477 may penetrate the blood-brain barrier (BBB) to inhibit intracranial glioma growth in mice. In conclusion, our study revealed that GNE-477 is likely to be a promising new drug for the molecular therapy of GBM.

## Materials and Methods

### Cell Culture

Human glioblastoma cells (U87 and U251) were obtained from the Institute of Biochemistry and Cell Biology, Chinese Academy of Sciences (Shanghai, China), and the ATCC version of the U87 cell line used in our study is most likely a glioblastoma, but its origin is unknown (https://web.expasy.org/cellosaurus/CVCL_0022). Cells were grown in an incubator at 37°C and with 5% CO2 in high-glucose Dulbecco’s modified Eagle’s medium (DMEM) supplemented with 10% fetal bovine serum and 1% penicillin/streptomycin (both from Gibco, Thermo Fisher Scientific).

### Drugs and Antibodies

**FIGURE 1:**
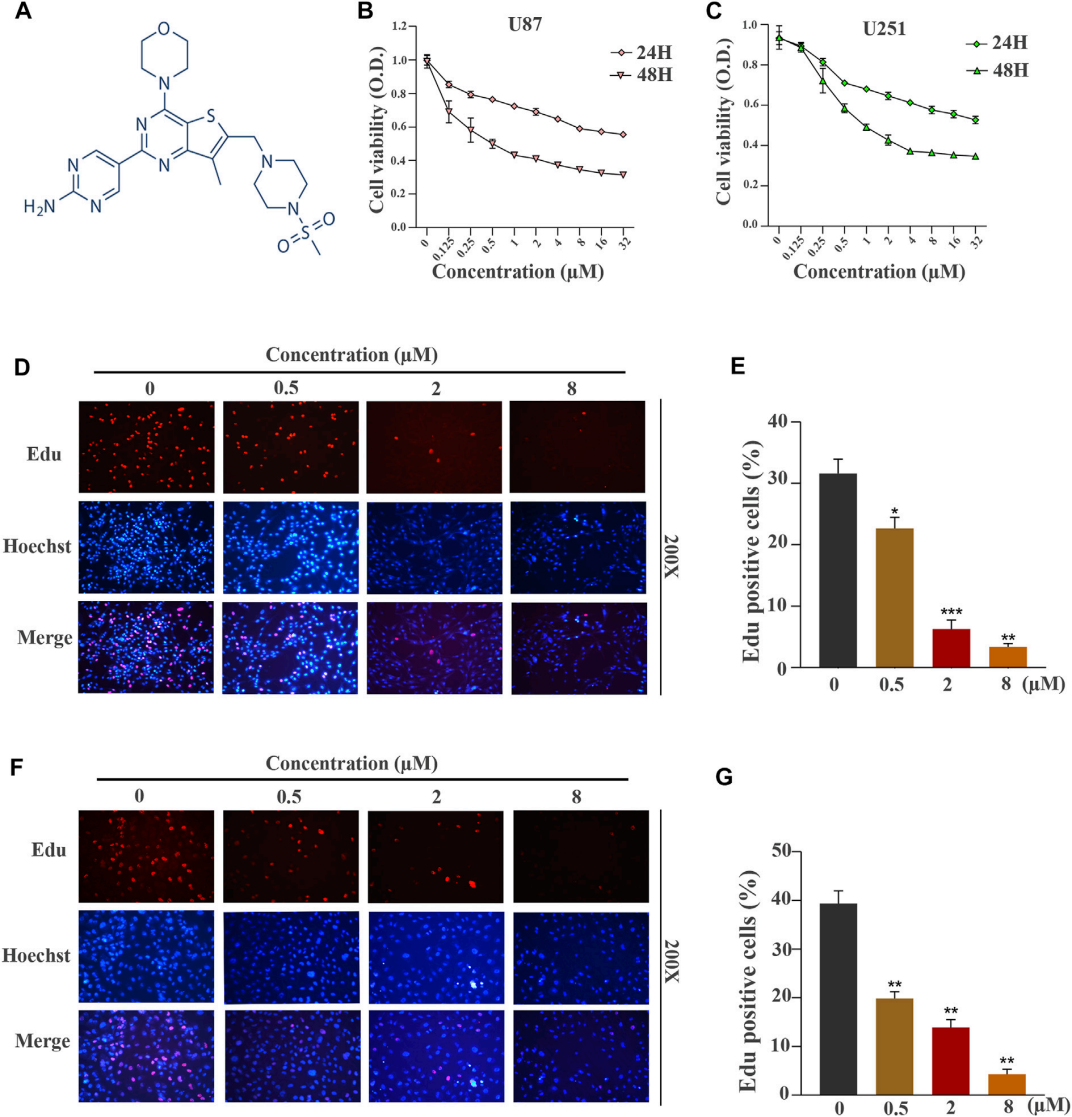
GNE-477 suppresses the proliferation of human GBM cells. **(A)** The molecular structure of GNE-477. **(B,C)** The viability of U87 and U251 cells was determined using CCK-8 assay after treatment with various concentrations of GNE-477 (0, 0.125, 0.25, 0.5, 1, 2, 4, 8, 16 or 32 µmol/L). **(D,F)** An EdU assay was performed to determine the inhibitory effect of GNE-477 on cell proliferation. The nuclei were stained with Hoechst (blue), and the proliferating cells were stained with EdU (red). **(E,G)** Data are reported for at least three independent experiments. *p < 0.05, **p < 0.01, and ***p < 0.001.

GNE-477 [S6516, purity (98.43%)] (Figure 1A) and the AKT phosphorylation activator SC79 (S7863, purity (>97%)) were both purchased from Selleckchem (Houston, TX, United States), and GNE-477 was dissolved and preserved in dimethyl sulfoxide (DMSO) obtained from Servicebio (G5051, Wuhan, China). The following antibodies were used: anti-AKT (product no.4691, Cell Signaling Technology (CST), United States), anti-phospho-AKT (product no. 4060; CST, United States), anti-CyclinD1 (60,186-1- Ig, Proteintech, Wuhan, China), anti-MMP2 (10,373-2- AP, Proteintech, Wuhan, China), anti-Bax (50599-2-Ig, Proteintech, Wuhan, China), anti-Bad (product no. 9239,CST,United States), anti-Bcl-2 (127891-AP,Proteintech, Wuhan, China), anti-mTOR (20657-1-AP, Proteintech, Wuhan, China), anti-phospho-mTOR (ab109268, Abcam, United Kingdom), and anti GAPDH (60004-1-Ig, Proteintech).

### Cell Viability Assay

The antitumor activity of GNE-477 was determined using a Cell Counting Kit-8 (CCK-8) assay, which was operated according to the protocol provided by the supplier (Dojindo, Shanghai, China). U87 and U251 cells were seeded in 96-well plates at a density of 5,000 cells per well. When the cells adhered and entered the logarithmic growth phase, they were treated with different concentrations of GNE-477 (0, 0.125, 0.25, 0.5, 1, 2, 4, 8, 16 or 32 µM) for 24 h or 48 h. Then, 10 µL of CCK-8 solution were added to each well and incubated at 37°C for 1 h. Finally, their OD values at 450 nm were measured with a plate reader. Each group was subjected to three independent assays.

### EdU-DNA Synthesis Assay

Cells in logarithmic growth phase were seeded in 96-well plates at a density of approximately 6 × 10^3^ cells per well and cultured until reaching the normal growth phase. Then, U87 and U251 cells were treated with different concentrations of GNE-477 (0, 0.5, 2 or 8 µM) for 48 h. According to instructions of the Cell-Light EdU Apollo567 *In Vitro* Kit (C10310-1, RiboBio, Guangzhou, China) provided by the supplier, 100 µL of 50 µM EdU medium was added and cells were cultured at 37°C in the presence of 5% CO2 for 2 h, and then the cell fixative containing 4% paraformaldehyde was incubated with the cells for 30 min at room temperature. Each well was then neutralized with 50 µL of glycine to neutralize excess aldehyde groups and incubated in 100 µL of a Triton X-100 decolorization solution on a shaker for 10 min. Finally, 100 µL of 1X Apollo® reaction cocktail was added and incubated for 30 min, followed by counterstaining with 1X Hoechst 33,342 for 30 min. Fluorescence images of EdU and Hoechst 33,342 were observed using a fluorescence microscope (BX51, Olympus, Japan).

### Cell Cycle Distribution Analysis

Flow cytometry combined with a cell cycle kit (KGA512, KeyGEN BioTECH, Nanjing, China) were used to analyze the cycle distribution of U87 and U251 cells. Cells were seeded into 6-well plates, treated with different concentrations of GNE-477 (0, 0.5, 2 or 8 µM) for 48 h, digested with 0.25% trypsin and collected by centrifugation. Cells were washed with PBS once and fixed with 70% ice-cold ethanol, then moved to −20°C and incubated overnight. The fixative was removed by rinses with PBS before staining, and 500 µL of a preprepared PI/RNase A working solution were added and incubated in the dark for 30 min at room temperature. The final results were measured using a CytoFLEX flow cytometer (Beckman Coulter), and data were further analyzed using FlowJo 10.0.7 software.

### Wound-Healing Assay

Cells were seeded in 6-well plates and cultured until the cell density reached approximately 70–80%. A straight scratch was slowly generated in the cell monolayer with a yellow pipette tip. After removing the floating cells by washing the well with PBS three times, serum-free medium mixed with GNE-477 (0 and 0.08 µM) was added and incubated with the cells at 37°C and 5% CO2. A microscope (Olympus BX51, Japan) was used to record scratch images of each group at the same location at 0, 24 and 48 h. ImageJ software was used to analyze the final distance of the healed scratch wound.

### Transwell Assay

Cell invasion experiments were performed using Transwell chambers and polycarbonate membranes with pore sizes of 8.0 µm U87 and U251 cells treated with 0, 0.02, 0.04 or 0.08 µM GNE-477 for 24 h were inoculated into the top chambers at a density of 8,000 cells per well, and 200 µL of serum-free medium were added to the upper chamber, while 600 µL of DMEM containing 10% fetal bovine serum were added to the corresponding lower chamber. After 24 h of incubation in a 37°C and 5% CO2 incubator, the cells were fixed with 4% paraformaldehyde for 30 min and then stained with 0.5% crystal violet for 30 min at room temperature. The cells remaining in the upper chamber were gently removed with a clean cotton swab. The chamber was dried naturally, and the results were observed and recorded under an inverted microscope at a magnification of 200X (Olympus BX51, Japan).

### Apoptosis Assay

The apoptotic rates of U87 and U251 cells were detected using an Annexin V-PE/7-AAD kit (Becton Dickinson, New Jersey, United States). Cells were seeded in 6-well plates and treated with 0, 0.5, 2 or 8 µM GNE-477 for 48 h. After digestion with EDTA-free trypsin, cells were collected by centrifugation at 4°C. Subsequently, the cells were washed twice with PBS precooled at 4°C, 100 µL of 1x binding buffer were added to resuspend the cells, and the density was adjusted to 1 × 10^6^ cells/ml. Then, 5 µL of Annexin V/PE and 10 µL of 7-AAD were added, mixed gently and incubated with the cells at room temperature for 15 min in the dark. Then, 400 µL of 1 × Binding Buffer were added to each tube. Finally, apoptotic cells were detected using a FACS Calibur flow cytometer (BD Biosciences, United States), and the results were statistically analyzed using FlowJo software (Tree Star, Ashland, OR, United States).

### Mitochondrial Membrane Potential (ΔΨm) Assay

The attenuation of ΔΨm is a characteristic event in the early apoptosis of cells. We performed JC-1 staining (Yeasen, Shanghai, China) according to the manufacturer’s protocol and analyzed the results with an Olympus BX51 microscope (Olympus, Japan). The cells were still seeded in 6-well plates and treated with or without GNE477 for 48 h. The ratio of red/green fluorescence intensity recorded based on final microscopic photographs reflects the loss of ΔΨm.

### Western Blot Analysis

U87 and U251 cells treated with 0, 0.5, 2 or 8 µM GNE-477 for 24 h were first gently washed twice with PBS and then lysed on ice with RIPA lysis buffer (Beyotime, China) for approximately 30 min, followed by centrifugation at 1.3 × 10^5^ rpm for 15 min. The protein concentration was measured using the BCA method (Beyotime, China). Subsequently, the lysate was mixed with the loading buffer at a certain ratio and boiled at 100°C for 10 min. Equal amounts of proteins were sequentially added to the wells of 10% or 12% SDS-PAGE gels for electrophoretic separation and then transferred to PVDF membranes (Millipore, Germany). PVDF membranes were then blocked with 5% skim milk for 1 h at room temperature and incubated with primary antibodies (P-AKT, AKT, CyclinD1, Bad, Bax, Bcl-2, MMP2, mTOR, P-mTOR and GAPDH) overnight at 4°C at the dilution ratio provided by the supplier. Subsequently, goat anti-rabbit or goat anti-mouse IgG secondary antibodies labeled with Alexa Fluor 680/790 were incubated with the membrane for 1 h. Finally, the results were visualized and analyzed using the Li-COR Odyssey infrared imaging system (Li-COR Biosciences, Lincoln, NE, United States).

### Immunofluorescence Staining

The passaged cells were seeded onto presterilized slides, placed in 6-well plates and cultured in complete medium. Forty-eight hours after GNE-477 treatment, cells were fixed with cell fixative containing 4% paraformaldehyde for 15 min. After permeabilization of cell membranes with 0.5% Triton X-100, cells were then blocked with 1% BSA for 30 min. The diluted anti-CyclinD1 antibody was incubated with the cells overnight at 4°C. The next day, the primary antibody was removed and then cells were incubated with an Alexa Fluor-labeled secondary antibody (Antgene, Wuhan, China). DAPI (ANT046, Antgene) was used to stain the nucleus. Automated microscopy (Olympus BX63; Japan) was used to capture images.

### Immunohistochemistry and HE Staining

Brain tissue specimens were immobilized in formalin and embedded in paraffin. The sections were deparaffinized, hydrated, and subjected to antigen retrieval in 10 mM sodium citrate (pH, 6.0). After an incubation with 3% H2O2 for 10 min, sections were blocked with serum for 1 h and then incubated overnight with the primary antibody at 4°C. After washes with cold phosphate-buffered saline (PBS), the secondary antibody was incubated with the sections at room temperature for 1 h. Finally, the tissue sections were stained with DAB (Servicebio), followed by hematoxylin counterstaining. HE staining was performed according to standard procedures. Images were visualized using an Olympus BX51 microscope (Olympus).

### Intracranial Xenograft Model

All 20 6-week-old BALB/c nude mice used in this study were purchased from Shulapo Biotechnology Co., Ltd. (Wuhan, China). During stereotactic implantation, the nude mice were anesthetized with isoflurane inhalation. Then, 1 × 10^6^ luciferase-expressing U87MG cells suspended in 3 μL of PBS were slowly injected into the right striatum of the mice (3.5 mm from the midline of the brain and 2 mm in front of the coronal suture). The needles were left in place for 5 min before retraction. The skull cavity was closed with bone wax, and the wound was sutured immediately. After 7 days, 20 mice were randomly divided into two groups (*n* = 10). Nude mice in the experimental group were injected with GNE-477 (25 mg/kg) through the tail vein every other day 7 consecutive times, while mice in the control group were injected with the same volume of DMSO. On the 30th day after intracranial tumor implantation, five mice from each group were randomly selected to evaluate tumor growth using the Living animal bioluminescence imaging system, and the bioluminescence value of tumors was quantitated using the Living Image 2.5 software package (Xenogen). Then, the mice were euthanized through cervical dislocation. The brain was removed, fixed, embedded in paraffin and sectioned for hematoxylin and eosin (H&E) staining and immunohistochemistry. The remaining mice were euthanized at the same time, and their brains were dissected to remove fish-like tumor tissue for Western blotting analysis. This study was approved by the Committee of Animal Care and Use of Renmin Hospital of Wuhan University.

### Statistical Analysis

All data were analyzed using GraphPad Prism 8.0 software. Three independent experiments were carried out, and the results are reported as the means ± standard deviations (SD). The unpaired Student’s t-test was used to compare the differences between two groups, while one-way analysis of variance was used for comparisons between three or more groups. A *p* value of less than 0.05 was considered statistically significant.

## Results

### GNE-477 Suppresses the Proliferation of Human Glioblastoma Multiforme Cells

The chemical structure of a new type of small-molecule PI3K/mTOR dual inhibitor, GNE-477, is shown in Figure 1A. We first performed CCK-8 experiments to explore whether this inhibitor is effective against glioblastoma. GNE-477 exerted an obvious inhibitory effect on U87 and U251 cells, and the effect on the viability of the two cell lines was dose-dependent (Figures 1B,C). At the same time, we observed that the IC50 values of GNE-477 for U87 and U251 cells were 0.1535 µmol/L and 0.4171 µmol/L, respectively. An EdU-DNA synthesis assay was performed to detect the number of EdU-positive cells after GNE-477 treatment and to further evaluate the inhibitory effect of GNE-477. The DNA replication levels of U87 (Figures 1D,E) and U251 (Figures 1F,G) cells treatment were much lower after GNE-477 than those after an equivalent DMSO treatment. With an increasing GNE-477 concentration, the DNA replication of U87 and U251 cells was inhibited to a greater extent. Based on the aforementioned results, GNE-477 possesses antiglioma properties.

### GNE-477 Arrests the Cell Cycle at G0/G1 Phase in Glioblastoma Multiforme Cells

**FIGURE 2:**
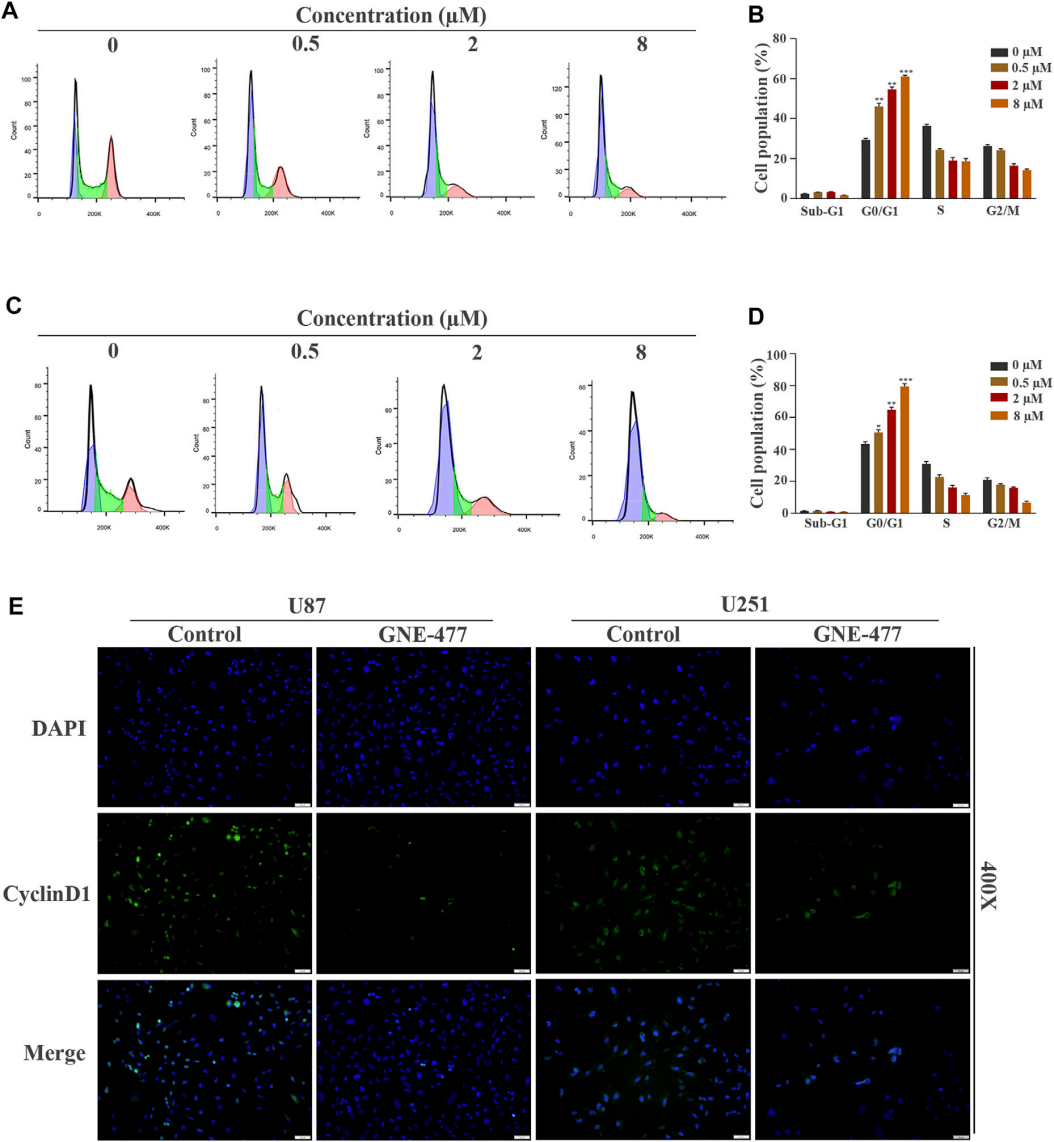
GNE-477 arrests the cell cycle of GBM cells in G0/G1 phase. **(A–D)** The cell cycle distribution of U87 and U251 cells treated with different concentrations (0, 0.5, 2, or 8 µmol/L) of GNE-477 for 48 h was detected using flow cytometry, and the percentage of cells at each stage was statistically analyzed in at least three independent experiments. *p < 0.05, **p < 0.01, and ***p < 0.001. **(E)** Immunofluorescence images of U87 and U251 cells confirmed the expression of CyclinD1.

Cell proliferation is one of the important characteristics of cell life, and the cell cycle is closely related to cell proliferation (Alenzi, 2004). Therefore, we hypothesized that GNE-477 blocked the cell division cycle of glioma cells. U87 and U251 cells were cultured with medium containing different concentrations of GNE-477 for 48 h, and flow cytometry was used to analyze the distribution of the cell cycle. Compared with the control group, the percentage of G1 phase cells increased continuously with increasing drug concentration, while the sum of S phase and G2 phase cells decreased gradually (Figures 2A–D). This result corresponded with the number of EdU-positive cells. The cell cycle is tightly regulated by cyclins and their catalytic partners, cell cycle-dependent kinases (CDKs) (Bertoli et al., 2013; Pérez-Posada et al., 2020). We performed immunofluorescence and Western blotting to detect the expression level of the CyclinD1 protein, the key checkpoint of the cell cycle, and to explore the specific mechanism of G1 phase blockade by GNE-477. As expected, the expression level of CyclinD1 was negatively correlated with the GNE-477 treatment concentration (Figure 2E and Figure 5).

### GNE-477 Inhibits the Migration and Invasion of U87 and U251 Cells

**FIGURE 3:**
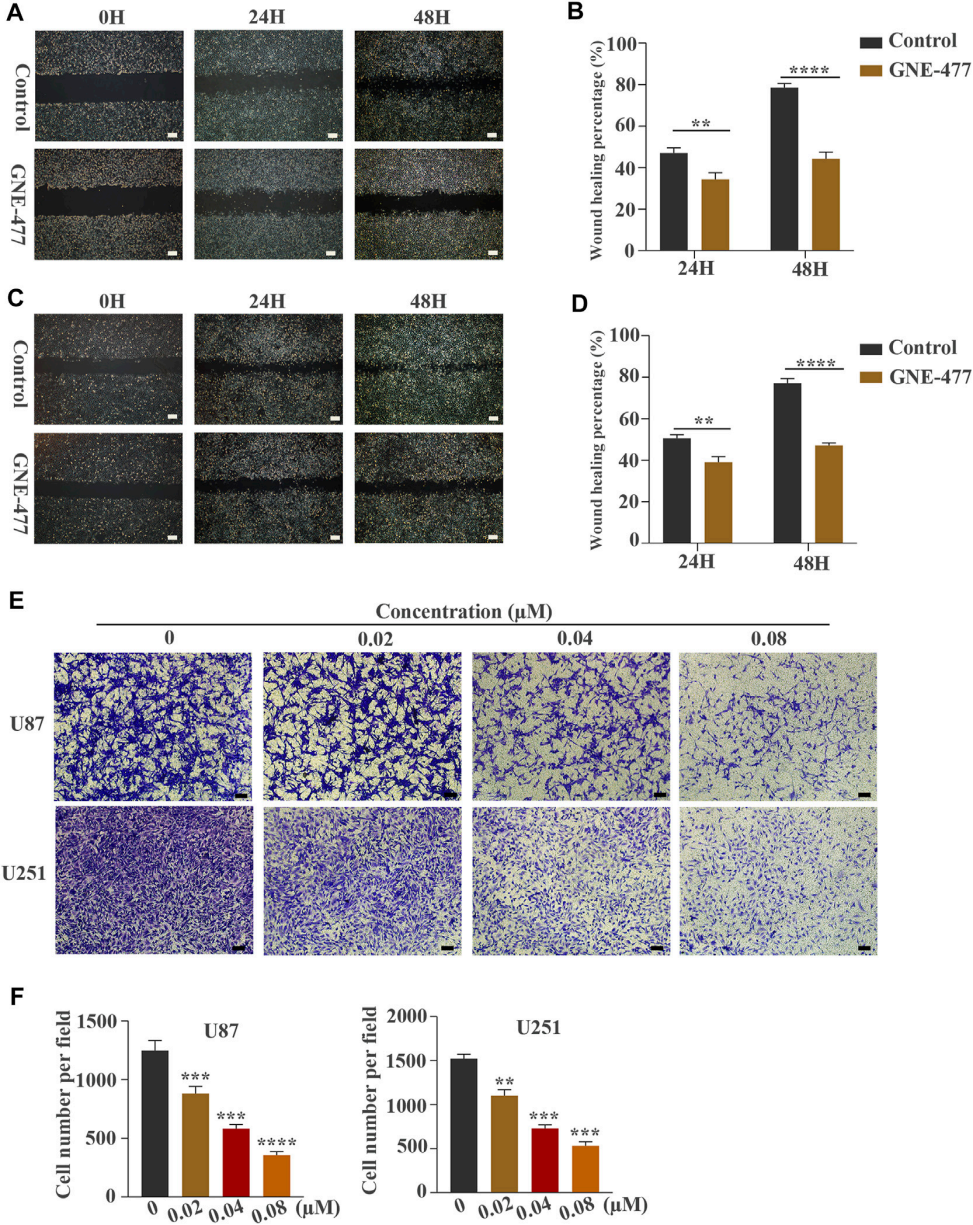
GNE-477 inhibits the migration and invasion of U87 and U251 cells. **(A–D)** The migration of the two cell lines was determined using wound-healing assays. The experimental group was treated with GNE-477, and the control group was treated with DMEM containing the same amount of DMS. Scale bars, 200 µm. **(E,F)** Transwell assays showed that GNE-477 exerted a significant inhibitory effect on the invasion of U87 and U251 cells, Scale bars, 200 µm. All data are presented as the mean values ±SD of experiments performed in triplicate; *p < 0.05, **p < 0.01, and ***p < 0.001 compared to the controls.

Scratch wound-healing assays and Transwell tests were used to detect the migration and invasion of glioblastoma cells as methods to examine the effect of GNE-477 on glioma metastasis. In this study, cells treated with DMSO alone were used as the control group. Compared with the nontreatment group, the GNE-477 treatment group showed a very slow recovery. After 24 and 48 h of growth, the spacing in the nontreatment group was significantly narrower and even basically healed (Figures 3A–D). At the same time, the invasion ability of U87 and U251 cells was also inhibited by increasing concentrations of GNE-477 (Figures 3E,F). These results were further confirmed by Western blot analysis. The expression of the MMP2 protein was significantly inhibited by GNE-477 in a dose-dependent manner (Figure 5).

### GNE-477 Induces Apoptosis in U87 and U251 Cells

**FIGURE 4:**
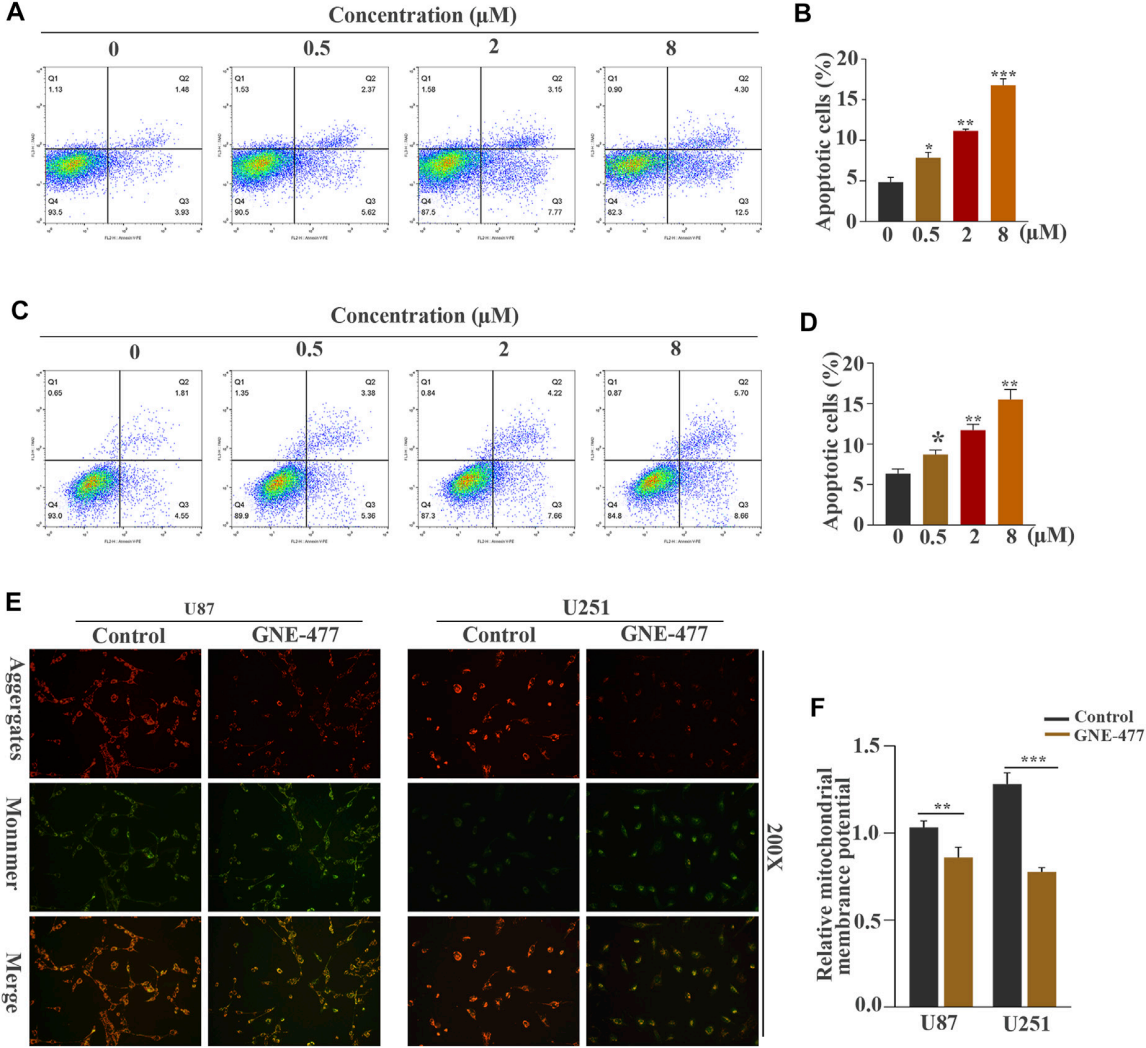
GNE-477 induces apoptosis in U87 and U251 cells. **(A–D)** Annexin V-PE/7-AAD staining combined with flow cytometry was used to analyze the apoptosis of U87 and U251 cells treated with different concentrations of GNE-477. **(E)** JC-1 staining showed changes in the ΔΨm of U87 and U251 cells cultured in the absence or presence of GNE-477 for 48 h. A decrease in the red (aggregates)/green (monomers) fluorescence intensity ratio indicates a loss of ΔΨm. All data are presented as the mean values ±SD of experiments performed in triplicate; *p < 0.05, **p < 0.01, and ***p < 0.001 compared to the controls.

**FIGURE 5:**
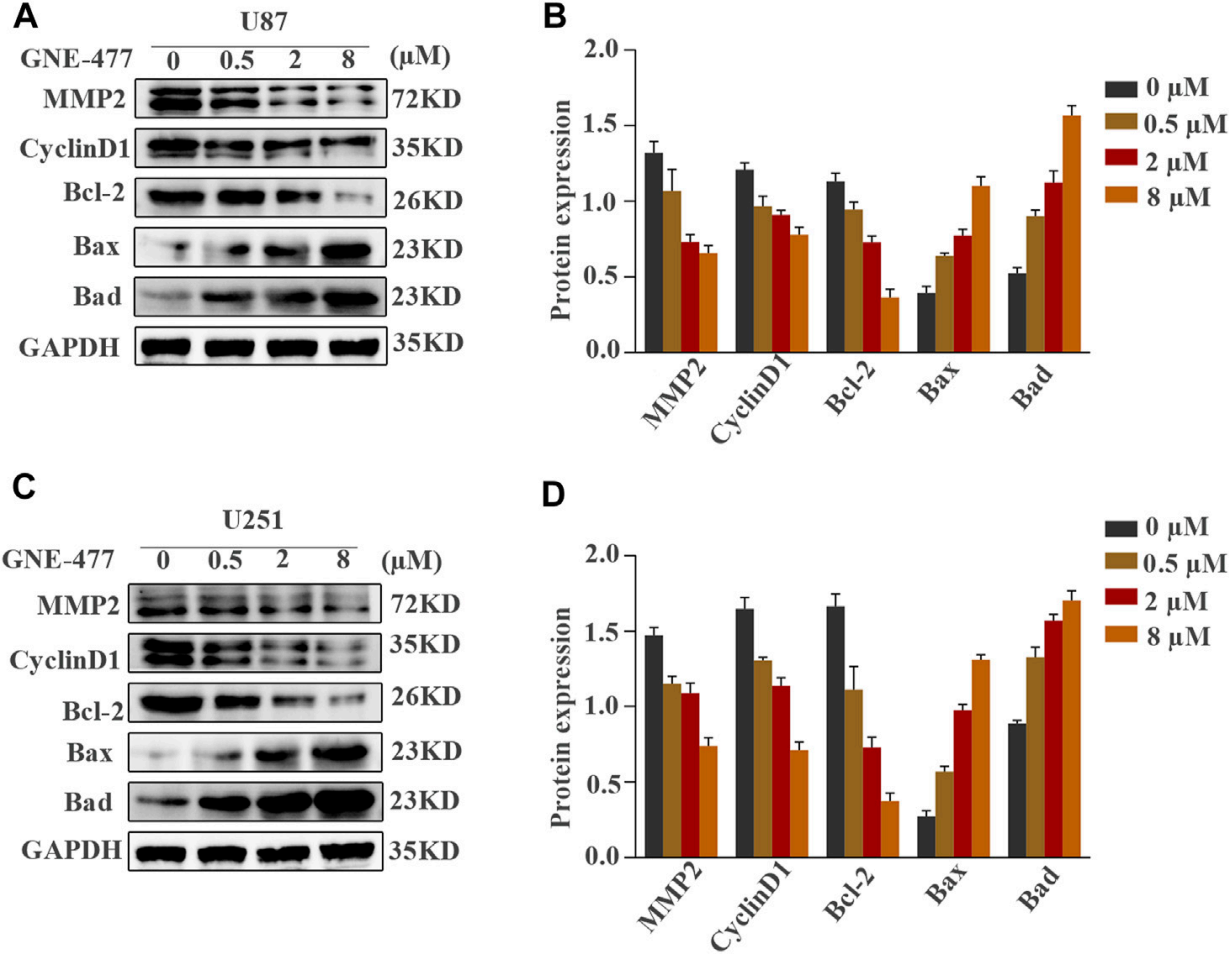
**(A,B)** Expression of the MMP-2, CyclinD1, Bcl-2, Bax, and Bad proteins in U87 and U251 cells treated with different concentrations of GNE-477 (0, 0.5, 2 or 8 µmol/L). GAPDH was used for normalization. **(C,D)** The data and graphs are representative of three independent experiments with similar results.

We performed a flow cytometry analysis of PE/7AAD staining to observe the effect of GNE-477 on cell apoptosis. An in-depth study of the changes in the apoptosis rates of U87 and U251 cells treated with different concentrations of GNE-477 was performed. The flow cytometry results showed that the rate of apoptosis continued to increase as the concentration increased (Figures 4A–D). Mitochondria play an indispensable role in apoptosis processes induced by various stimuli (Desagher and Martinou, 2000; Abate et al., 2020). Therefore, we further investigated whether the mitochondrial membrane potential was altered after GNE-477 treatment, as verified by JC-1 staining. Compared with the control group, GNE-477 substantially reduced the red-blue fluorescence ratio, reflecting the ΔΨm of the cells (Figures 4E,F). In addition, the Western blot results further showed significantly increased expression of the Bax and Bad proteins, while the expression of Bcl-2 was significantly reduced (Figure 5). These results indicate that GNE-477 induces apoptosis in U87 and U251 cells.

### The AKT/mTOR Signaling Pathway Is Blocked by GNE-477 in U87 and U251 Cells

**FIGURE 6:**
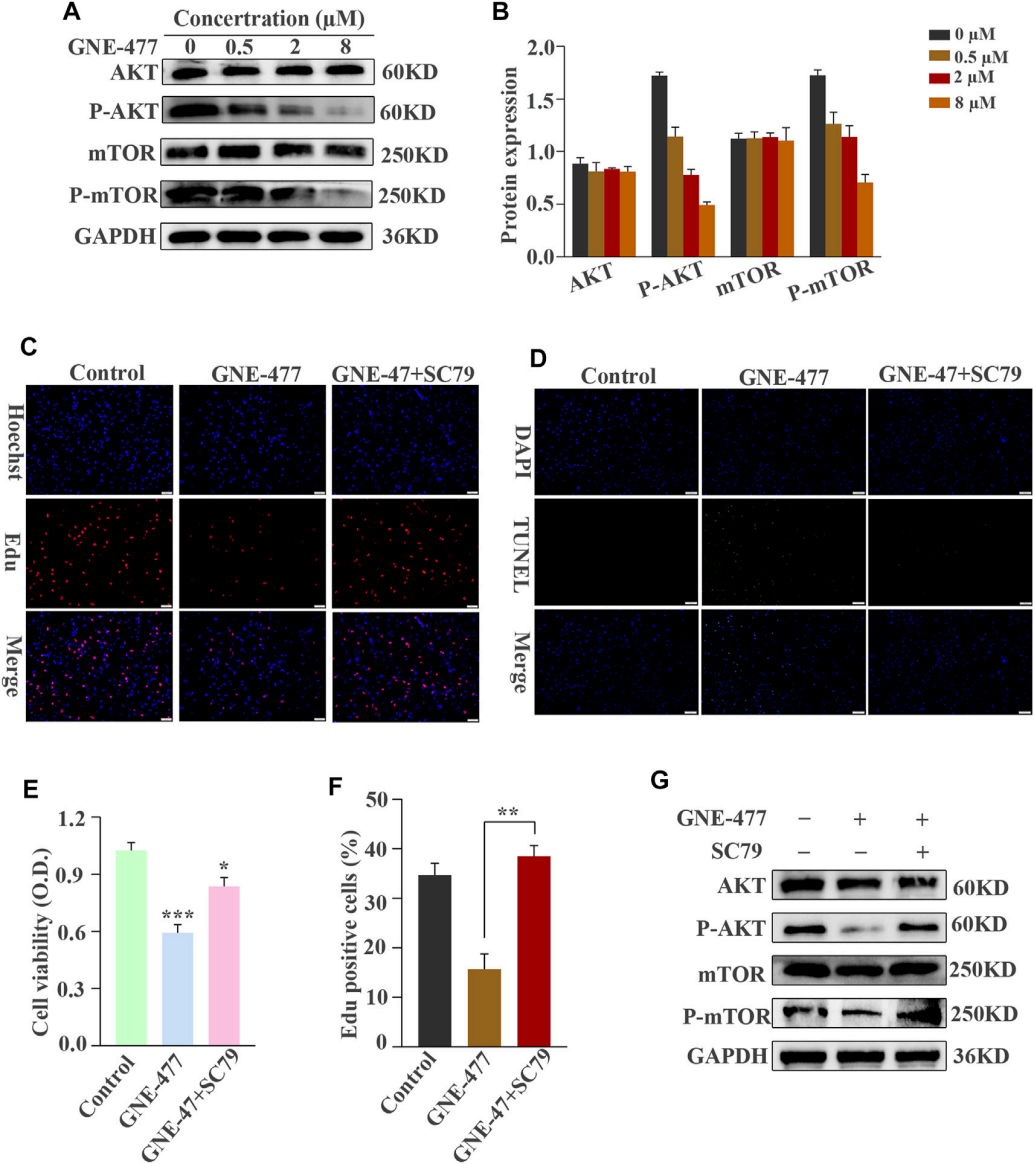
The AKT/mTOR signaling pathway is blocked by GNE-477 in U87 cells. **(A,B)** WB results were obtained after treating cells with various concentrations of GNE-477 or DMSO for 48 h **(C–F)** The AKT/mTOR signaling pathway is activated by SC79. EdU-DNA synthesis assays and CCK-8 assays were performed to investigate the proliferation of U87 cells. A TUNEL assay was performed to detect apoptosis in GBM cells. The results from at least three independent experiments are shown Scale bars, 50 µm. **(G)** Western blot analysis of AKT, P-AKT, mTOR, P-mTOR and GAPDH levels in GNE-477-pretreated U87 cells treated with SC79 (5 μg/ml). *p < 0.05, **p < 0.01, and ***p < 0.001.

As a dual inhibitor of PI3K/mTOR, GNE-477 exerts an obvious antitumor effect on glioma cells, prompting us to explore its possible mechanism. The level of the phosphorylated AKT protein decreased in cells treated with increasing concentrations of GNE-477 in a dose-dependent manner, while total AKT protein levels remained unchanged. A similar phenomenon was observed for mTOR, a downstream target of AKT, as total mTOR expression was unchanged and P-mTOR levels decreased in a dose-dependent manner (Figures 6A,B, and Supplementary Figures S1A,S1B). SC79 is an important agonist of AKT (Jo et al., 2012; Liu et al., 2018). In subsequent studies, SC79 was added to cells after 48 h of GNE-477 treatment. Notably, SC79 restored the levels of phosphorylated Akt and phosphorylated mTOR in GNE-477-treated cells (Figure 6G and Supplementary Figure S1G). Next, both the CCK-8 assay and EdU assay revealed that SC79 rescued GNE-477-mediated inhibition of cell proliferation (Figures 6C,E,F, and Supplementary Figures S1C,S1E,S1F), and the TUNEL assay indicated that SC79 rescued GNE-477-mediated promotion of apoptosis (Figure 6D and Supplementary Figure S1D). These results indicated that GNE-477 most likely inhibits glioma development by modulating the AKT/mTOR signaling pathway.

### GNE-477 Potently Inhibits Glioblastoma Multiforme Xenograft Tumor Growth in Mice

**FIGURE 7:**
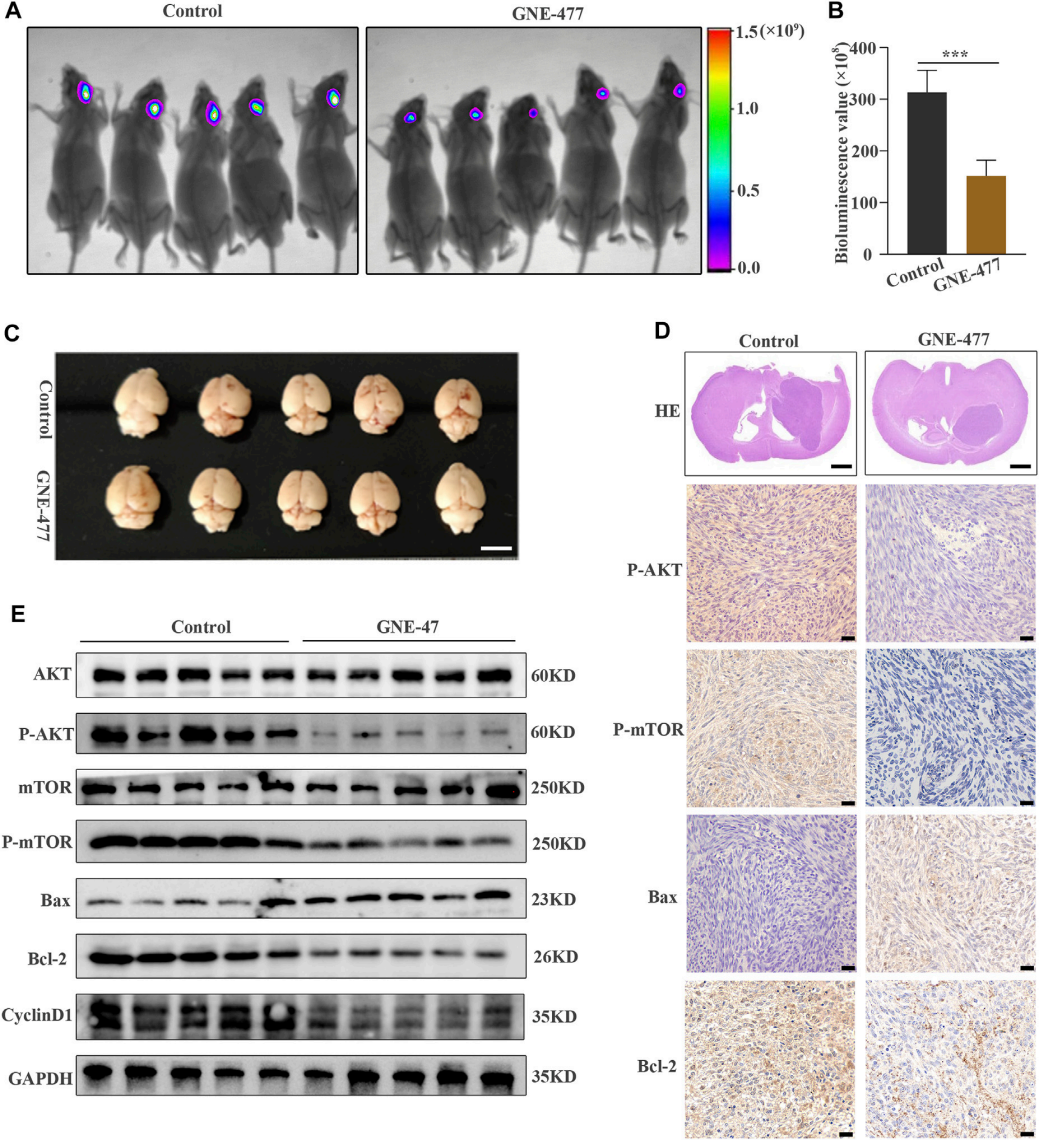
GNE-477 inhibits GBM cell proliferation in an intracranial xenograft model. **(A)** The bioluminescence imaging system assessed tumor growth 30 days after xenotransplantation and treatment with GNE-477 and DMSO. **(B)** Bioluminescence values were plotted statistically, with *p* values obtained for the comparison between the treatment group and the control group. **(C)** Five GNE-477-treated mice and five control mice were randomly selected from the experimental group and the control group. **(D)** Representative images of brain tissue sections from mice that received GNE-477 and DMSO are shown. Scale bars, 1.0 mm. **(E)** Western blot assay of the main protein expression levels in the control group and SC66-treated group. **(F)** Representative images of IHC staining for P-AKT, P-mTOR, Bax, and Cyclin D1. Scale bars, 20 µm *p < 0.05 and ***p < 0.001. The images are representative of three independent experiments with similar results.

The potential antitumor activity of GNE-477 *in vitro* and its inhibitory effect on an RCC animal tumor model greatly stimulated our research interest. Therefore, we used U87MG glioma cells expressing luciferase to establish an orthotopic glioma transplantation model. The volume of glioma in the nude mice treated with GNE-477 was significantly smaller than that in the control group, as evidenced both by *in vivo* bioluminescence imaging (Figures 7A,B) and by a visual observation of brain tumor tissues and HE staining (Figures 7C,D). Consistent with the *in vitro* results, Western blot analyses showed decreased levels of P-AKT, P-mTOR, Bcl-2 and CyclinD1 in the GNE-477-treated group, while the levels of the Bax and Bad proteins were increased (Figure 7E). Immunohistochemical staining also confirmed a similar trend (Figure 7F). Taken together, these data suggest that GNE-477 transits the BBB to a certain extent and effectively blocks glioma cell proliferation *in vivo*.

## Discussion

Glioblastoma (GBM) is the most common primary malignant brain tumor, accounting for 16% of all primary brain and central nervous system tumors (Thakkar et al., 2014). Various deep-seated causes have hindered the progress of GBM therapies (Shergalis et al., 2018), and thus far, it remains the most common and aggressive primary malignant brain tumor in adults (Paw et al., 2015). However, little progress has been achieved in improving the prognosis of patients with GBM in the past decade, which has prompted the development of more effective molecular targeted therapies. The PI3K/AKT/mTOR signaling cascade has long been implicated in regulating a variety of cellular processes, such as the proliferation, differentiation, survival, transformation and metastasis of tumor cells (Manning and Cantley, 2007; Ortega et al., 2020; Xu et al., 2020). At the same time, accumulating evidence suggests that GBM often exhibits overactivation of the PI3K-AKT pathway (Eyler et al., 2008; Molina et al., 2010). However, only a small number of PI3K inhibitors have entered clinical trials as treatments for GBM, as the blockade of a single pathway usually causes compensatory activation of other pathways to alleviate the inhibitory effect and maintain the survival of tumor cells (Eyler et al., 2008). More notably, mTOR plays an important role in regulating the growth, metabolism and protein synthesis of cancer cells, but even if the activities of PI3K and AKT are inhibited, cancer cells still maintain mTOR activation (Kharas et al., 2008), and this crosstalk and feedback between mTOR and PI3K greatly limit the therapeutic effects of mTOR or PI3K inhibitors. Therefore, dual inhibitors of PI3K/mTOR, such as NVP-BEZ235 (Yu et al., 2015) and XL765 (Zhao et al., 2019), have naturally attracted increasing interest from researchers.

Our aim was to identify new potential therapies for GBM. GNE-477, an effective dual PI3K/mTOR inhibitor, has shown satisfactory pharmacokinetic properties in rats, mice and dogs, as well as significant inhibition of PC3 tumor growth in other studies (Zhao et al., 2019). More importantly, GNE-477 has been reported to inactivate the PI3K-AKT-mTOR cascade by blocking the phosphorylation of p85, AKT1, p70S6K1 and S6, thereby inhibiting the growth of renal cell carcinoma *in vitro* and *in vivo* in nude mice (Ye et al., 2020). GNE-477 was more effective at inhibiting RCC cell survival and inducing apoptosis than other PI3K-AKT-mTOR inhibitors (LY294002, AZD 2014, and Perifosine) (Ye et al., 2020).

In the present study, we revealed that GNE-477, a dual PI3K/mTOR inhibitor, exerts antitumor effects on GBM by affecting multiple key cellular processes. First, GNE-477 reduced the viability of U87 and U251 cells in a dose-dependent manner, and the IC50 values of GNE-477-treated U87 and U251 cells were 0.1535 µmol/L and 0.4171 µmol/L, respectively. GNE-477 also significantly promoted G1 arrest and cell apoptosis in a dose-dependent manner. GNE-477 reduced the migration and invasion of human glioma cell lines in wound-healing and cell invasion experiments. Western blotting was performed to detect the expression of Bcl-2, Bax, Bad, MMP-2 and CyclinD1, and immunofluorescence staining was conducted to detect the expression of CyclinD1. At the same time, we observed a significant decrease in the level of phosphorylated AKT (Ser-473) in cells treated with GNE-477 in a dose-dependent manner, with the total AKT protein level remaining unchanged. We used the AKT phosphorylation agonist SC79 for rescue experiments to further verify whether the antitumor activity of GNE-477 was mediated by regulating the PI3K-AKT-mTOR signaling pathway and found that GNE-477 regulated this pathway in GBM cells. The effect was reversed by SC79.

A large number of new drugs used to treat diseases of the central nervous system often fail in clinical trials because they do not cross the BBB (Geldenhuys et al., 2015). However, our results indicate that GNE-477 has significant antitumor activity against intracranial GBM in our animal model compared to the control group. The therapeutic effects of GNE-477 were confirmed by bioluminescence imaging *in vivo*. The effects of compounds with the ability to cross the BBB have been reported, including lipid solubility, electric charge, tertiary structure, and degree of protein binding (Banks, 2009). Therefore, in a future experiment, we plan to further study the characteristics of the GNE-477 molecular structure and improve the efficiency of GNE-477 penetration through the BBB by encapsulating it in nanomaterials to determine whether we can target GNE-477 to intracranial tumor tissue, thus substantially improving the therapeutic effect of GNE-477.

Taken together, our results suggest that GNE-477-induced anti-GBM activity is largely due to inhibition of PI3K-AKT-mTOR signaling. Our study also reveals for the first time the antitumor effects of GNE-477 on GBM *in vitro* and *in vivo*, and our findings may contribute to its potential application as an effective treatment for GBM.

## Data Availability

The raw data supporting the conclusion of this article will be made available by the authors, without undue reservation.
