# Supplementary figures and images for: The New PI3K/mTOR Inhibitor GNE-477 Inhibits the Malignant Behavior of Human Glioblastoma Cells
Source: Front Pharmacol. 2021 Jul 26;12:659511. doi: 10.3389/fphar.2021.659511 (PMC8350478; doi:10.3389/fphar.2021.659511)

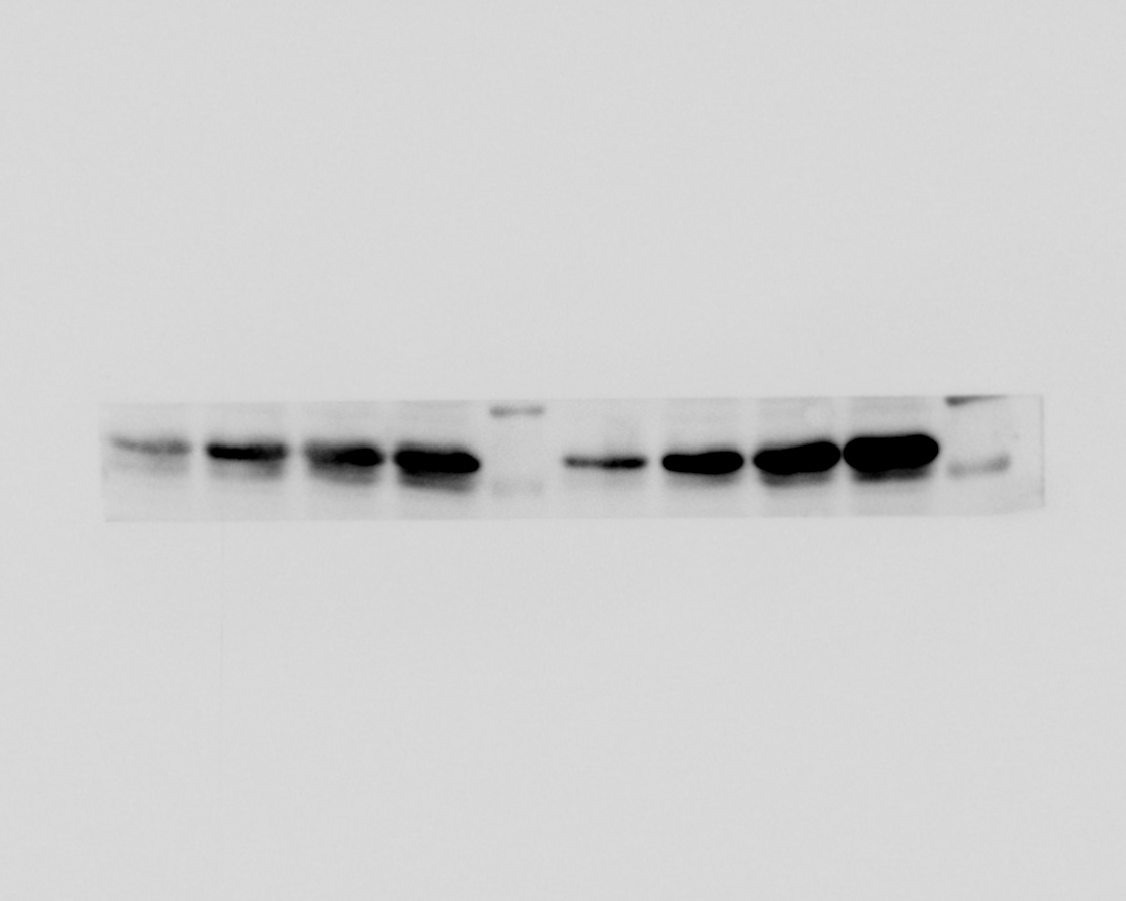

Supplement: Supplementary file 1 [file DataSheet1.zip › Original WB data/Fig 5-WB/A&C(bad).tif]

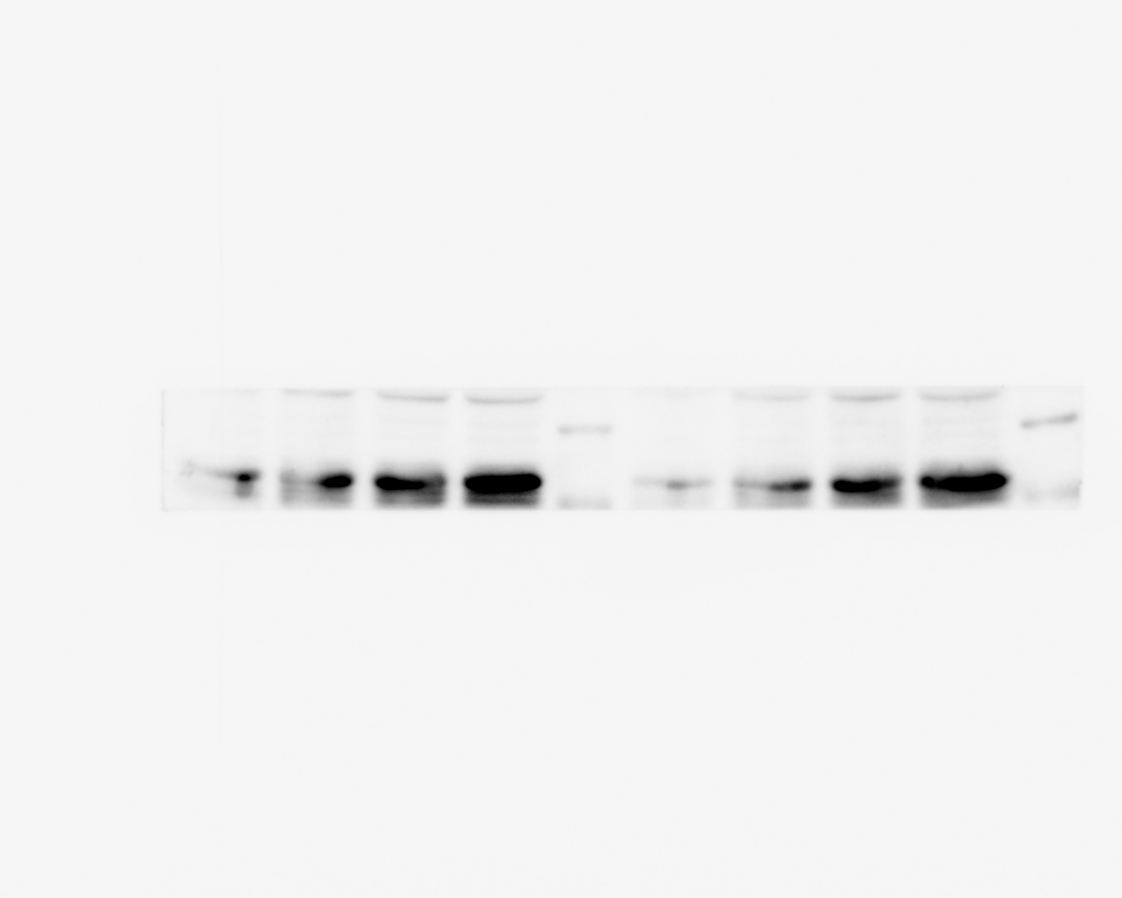

Supplement: Supplementary file 1 [file DataSheet1.zip › Original WB data/Fig 5-WB/A&C(bax).tif]

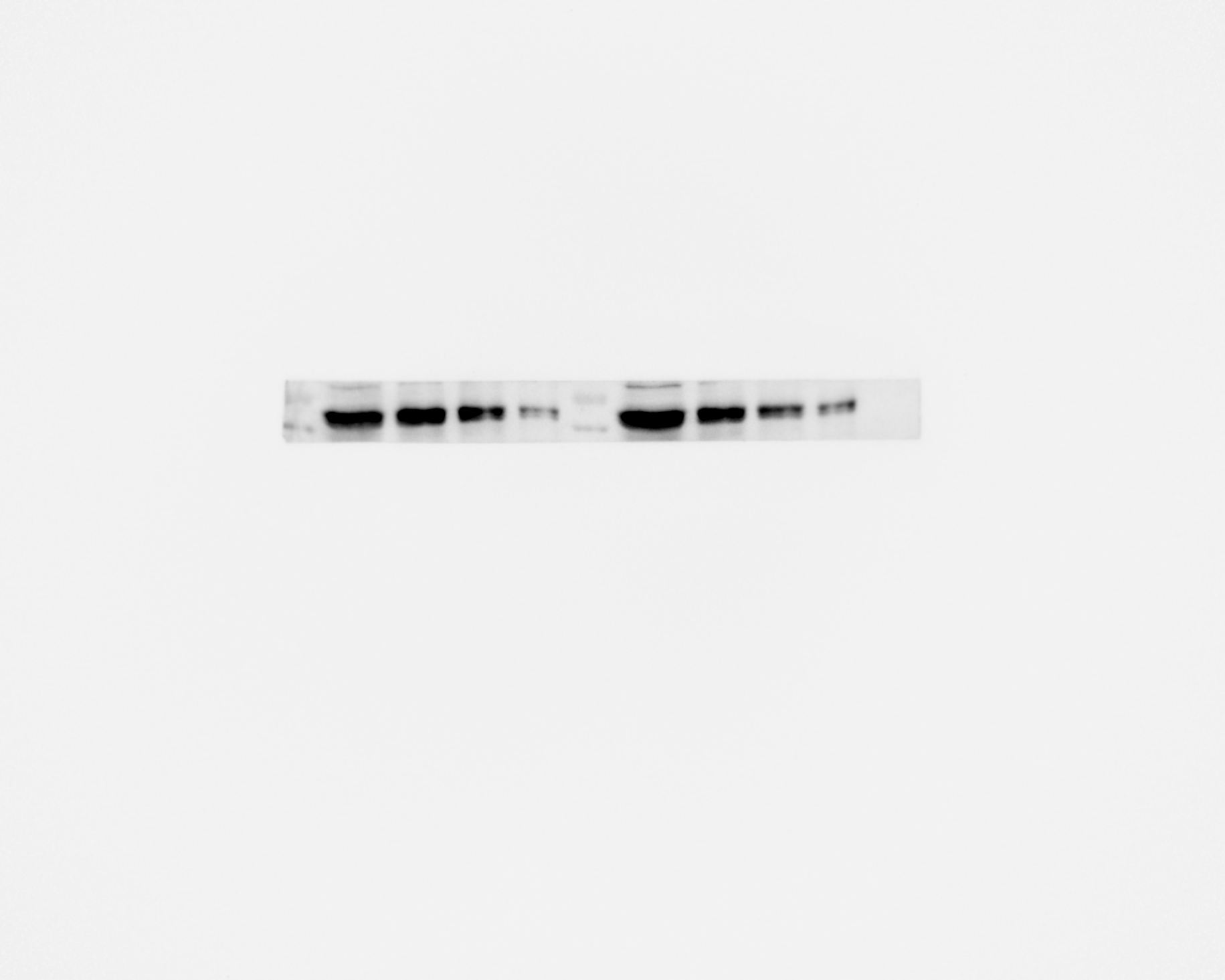

Supplement: Supplementary file 1 [file DataSheet1.zip › Original WB data/Fig 5-WB/A&C(bcl2).tif]

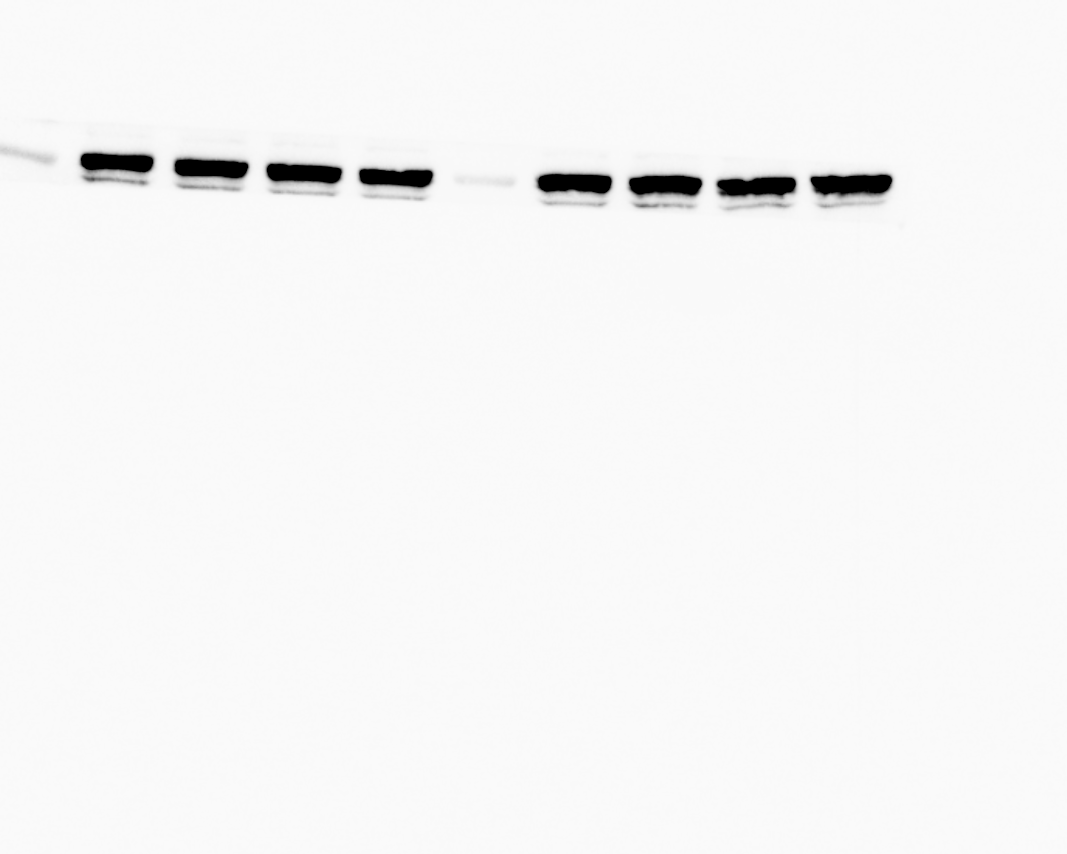

Supplement: Supplementary file 1 [file DataSheet1.zip › Original WB data/Fig 5-WB/A&C(GAPDH).tif]

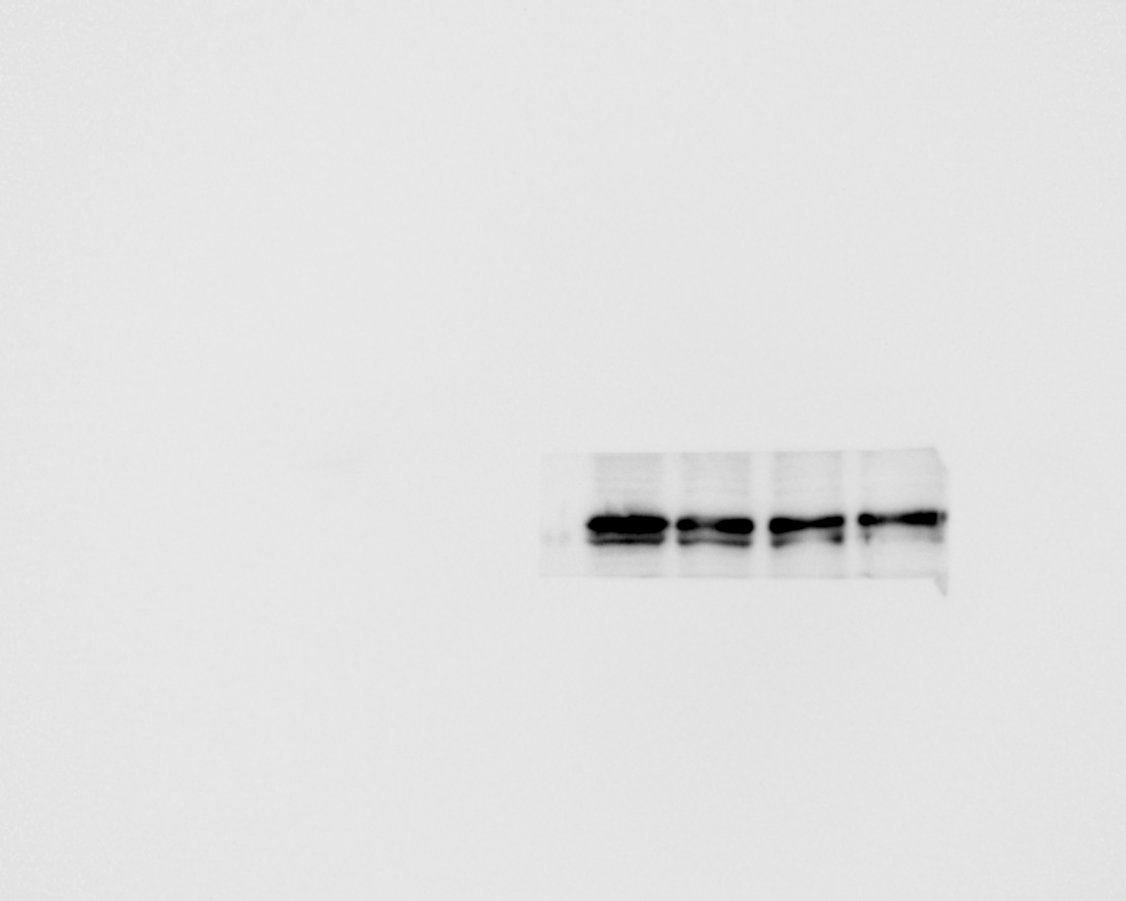

Supplement: Supplementary file 1 [file DataSheet1.zip › Original WB data/Fig 5-WB/A-Cyclin D1.tif]

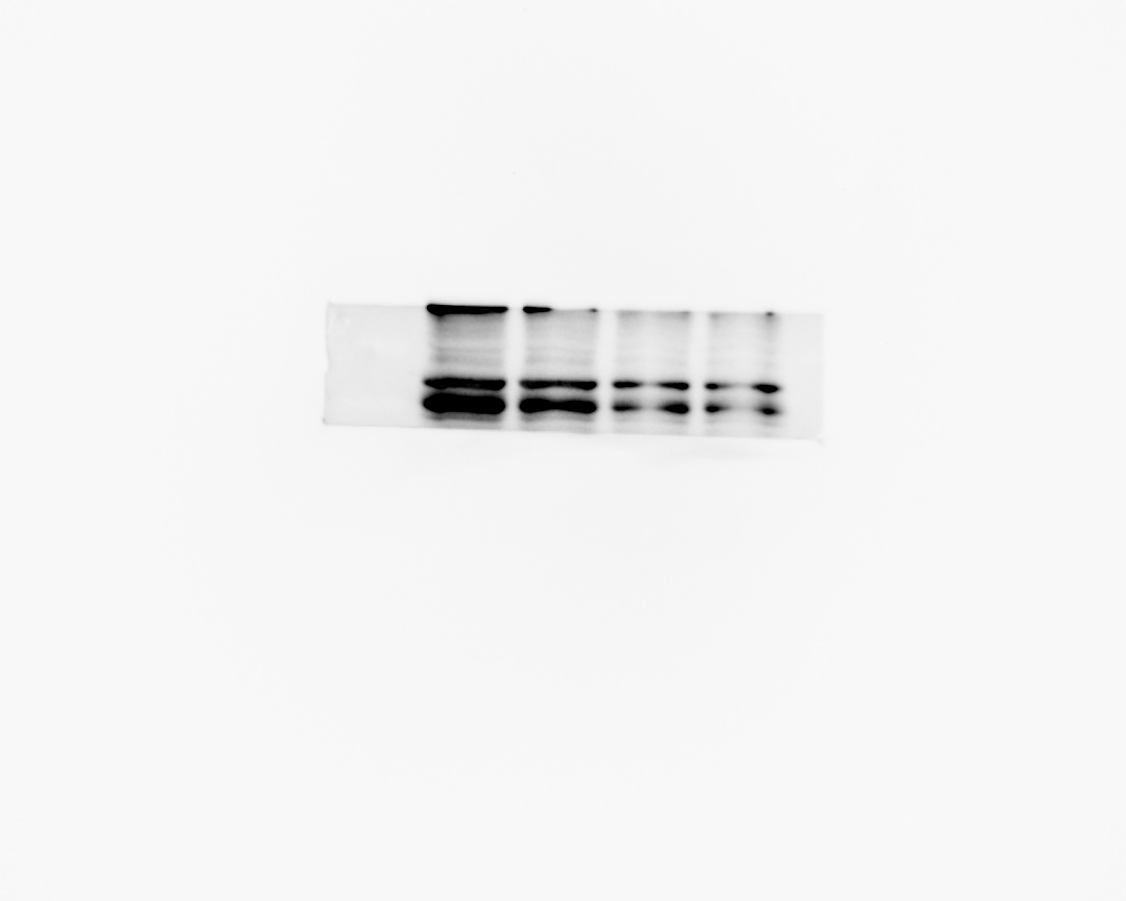

Supplement: Supplementary file 1 [file DataSheet1.zip › Original WB data/Fig 5-WB/A-MMP2.tif]

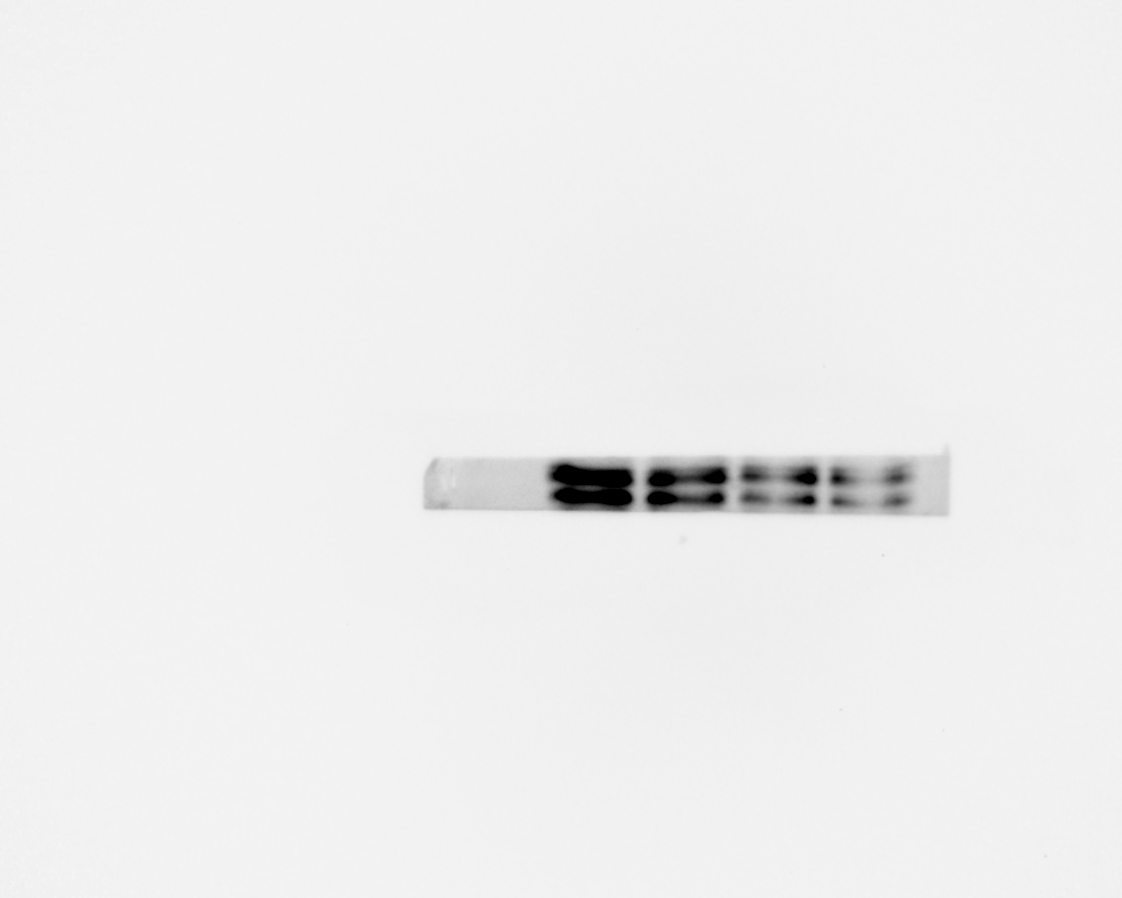

Supplement: Supplementary file 1 [file DataSheet1.zip › Original WB data/Fig 5-WB/C-Cyclin D1.tif]

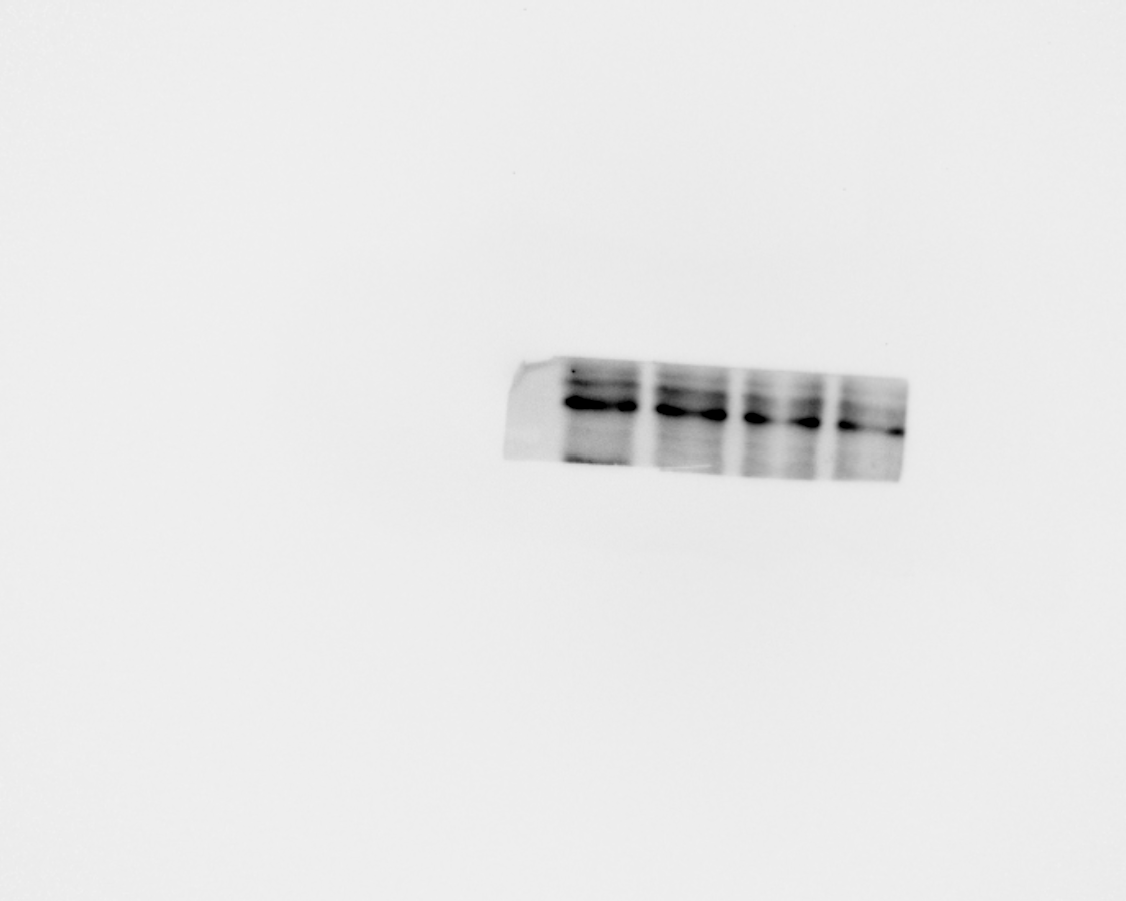

Supplement: Supplementary file 1 [file DataSheet1.zip › Original WB data/Fig 5-WB/C-MMP2.tif]

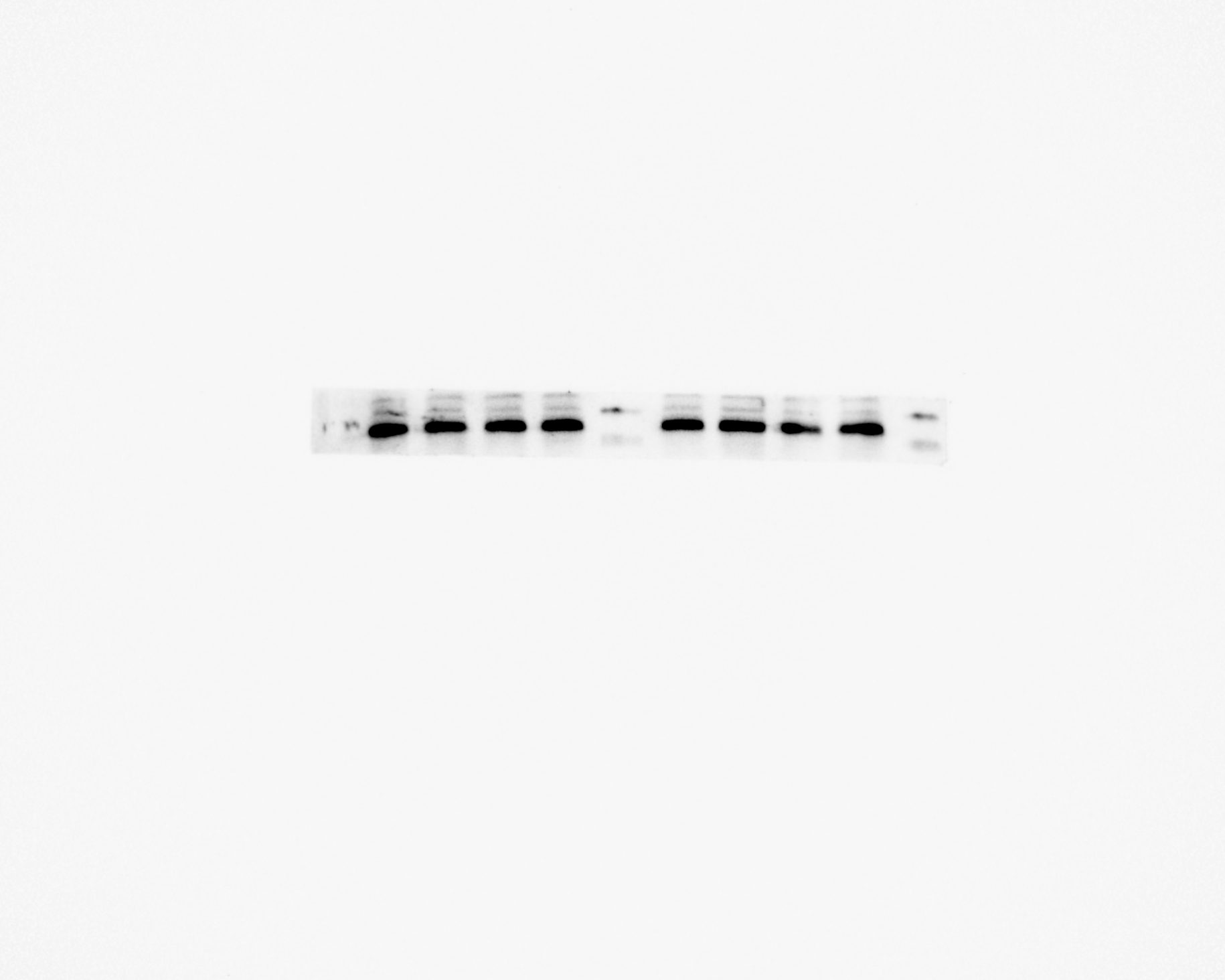

Supplement: Supplementary file 1 [file DataSheet1.zip › Original WB data/Fig 6&S1-WB/Fig6&S1-A(akt).tif]

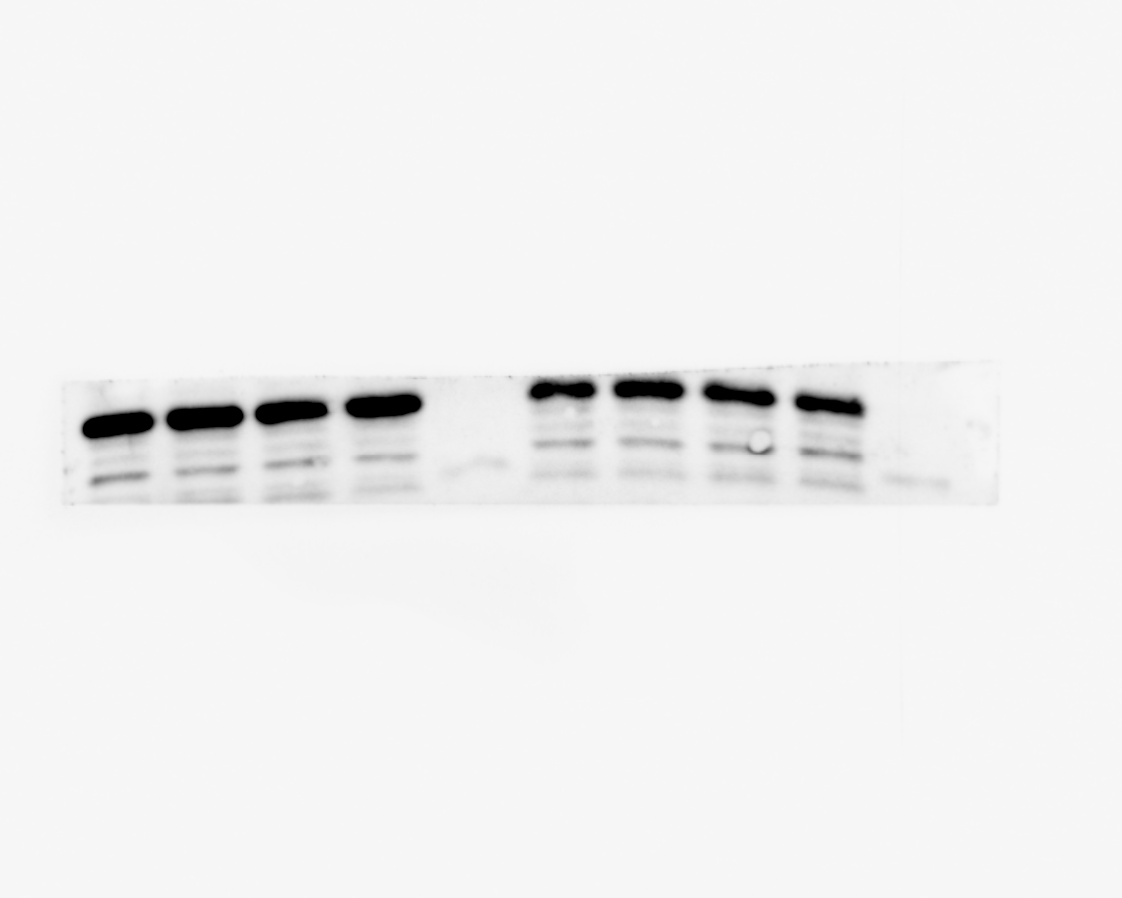

Supplement: Supplementary file 1 [file DataSheet1.zip › Original WB data/Fig 6&S1-WB/Fig6&S1-A(gapdh).tif]

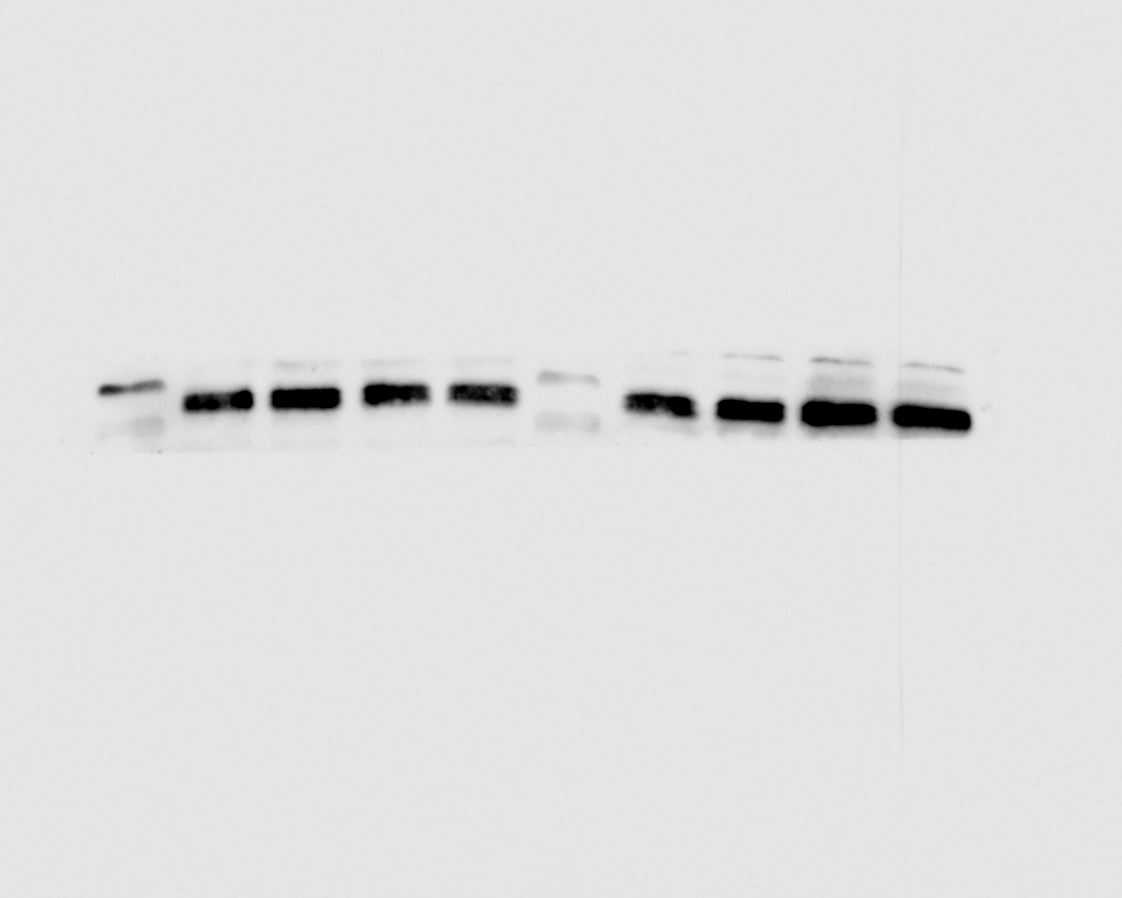

Supplement: Supplementary file 1 [file DataSheet1.zip › Original WB data/Fig 6&S1-WB/Fig6&S1-A(mTOR).tif]

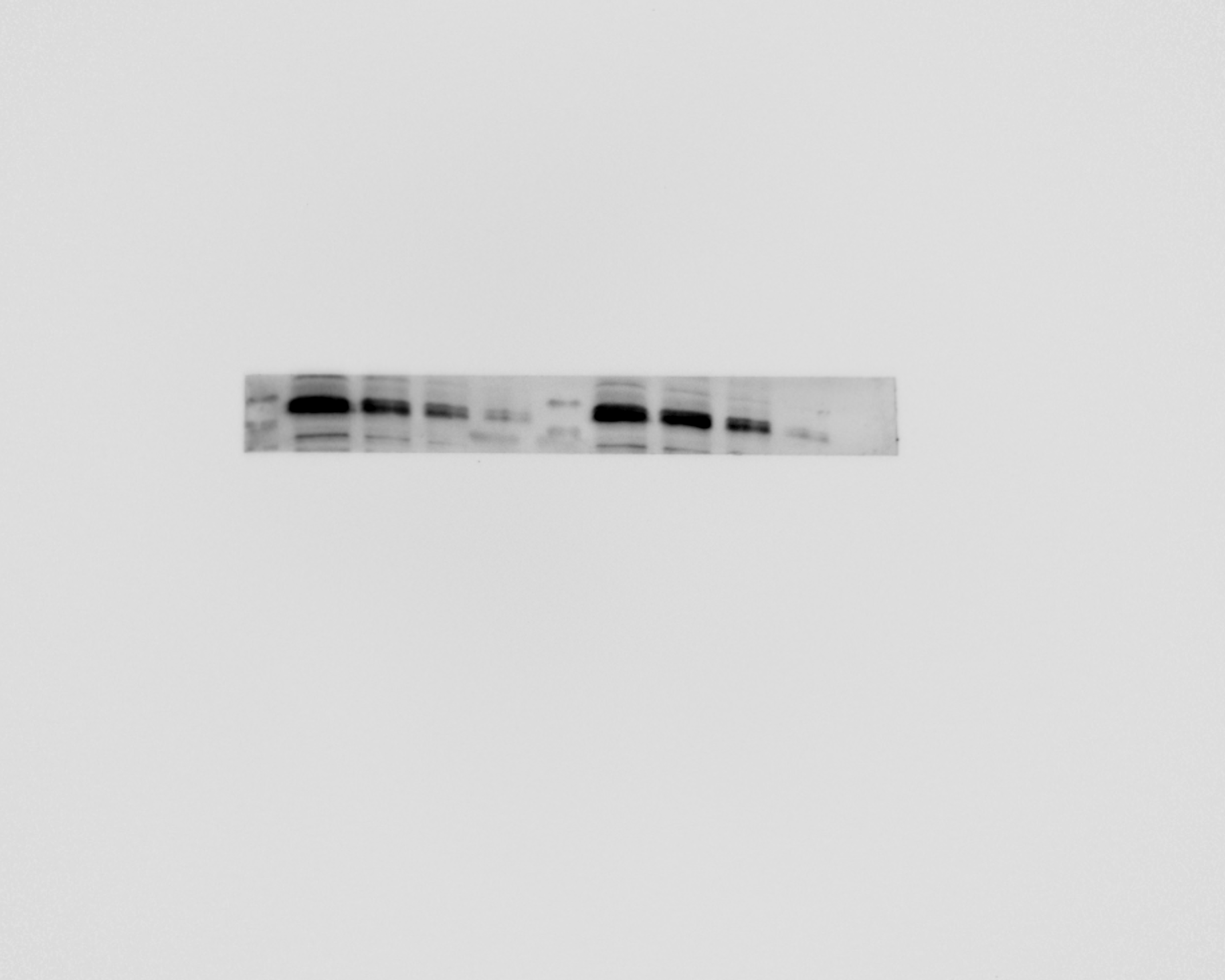

Supplement: Supplementary file 1 [file DataSheet1.zip › Original WB data/Fig 6&S1-WB/Fig6&S1-A(p-akt).tif]

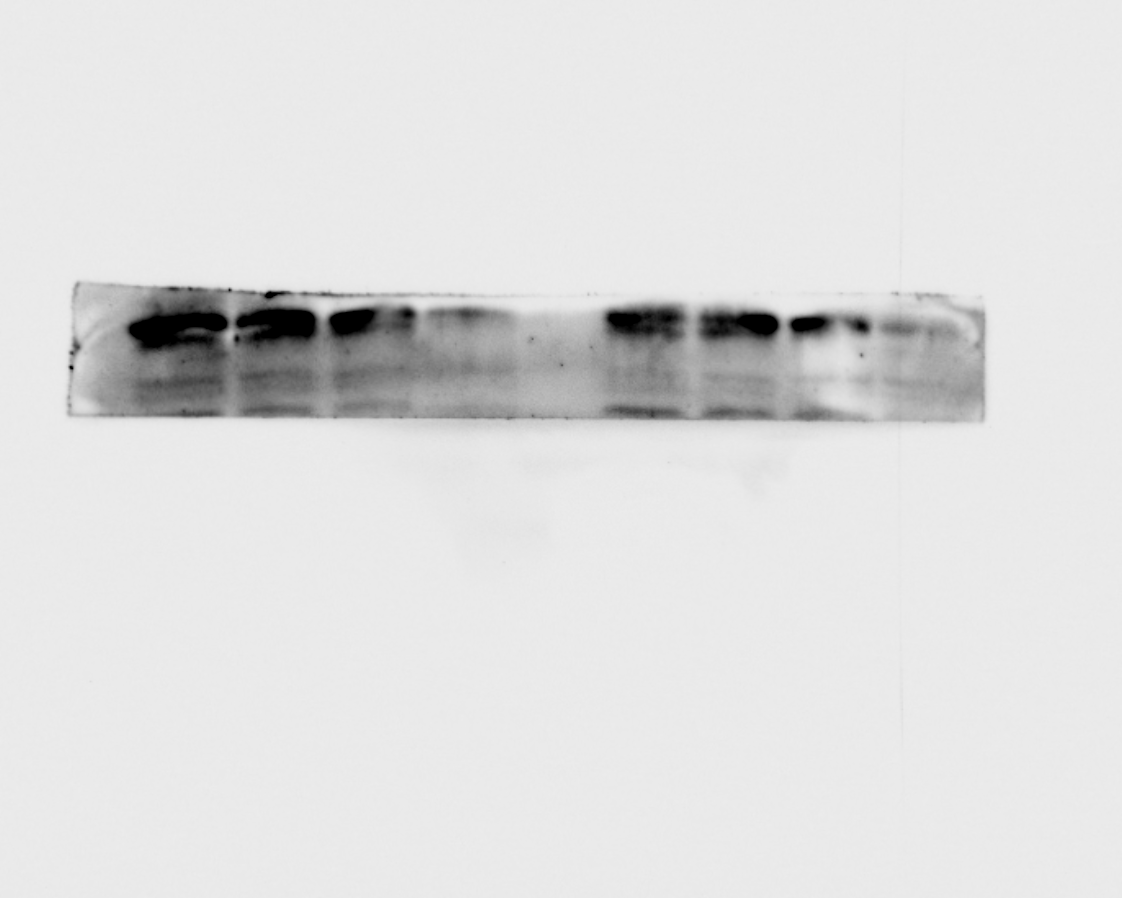

Supplement: Supplementary file 1 [file DataSheet1.zip › Original WB data/Fig 6&S1-WB/Fig6&S1-A(p-mtor).tif]

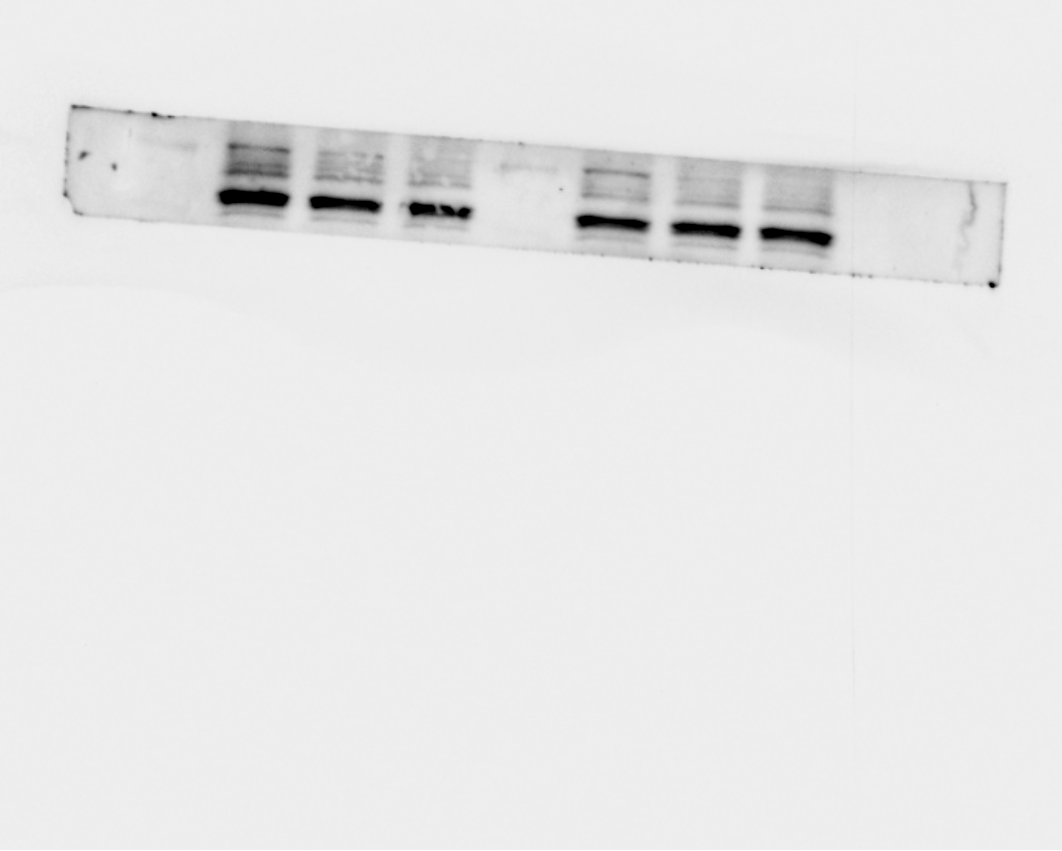

Supplement: Supplementary file 1 [file DataSheet1.zip › Original WB data/Fig 6&S1-WB/Fig6&S1-G(akt).tif]

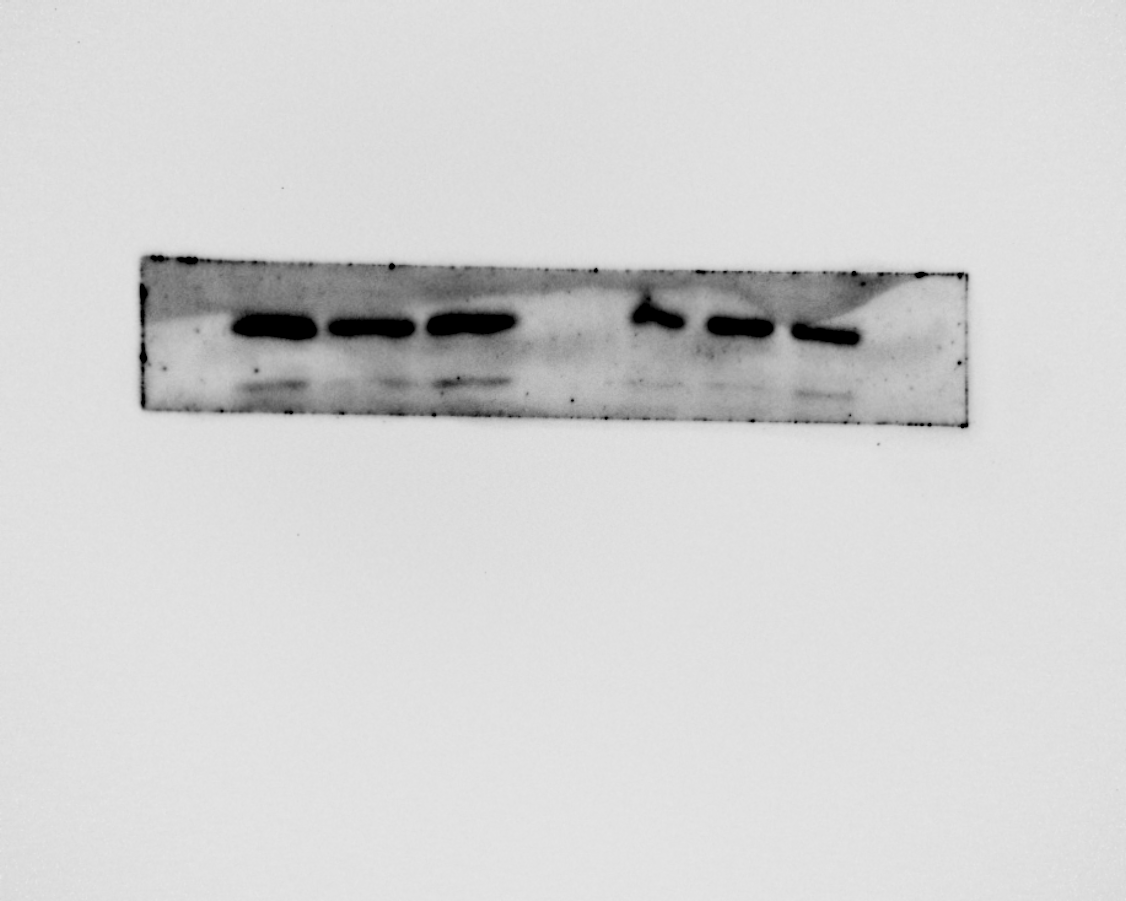

Supplement: Supplementary file 1 [file DataSheet1.zip › Original WB data/Fig 6&S1-WB/Fig6&S1-G(mtor).tif]

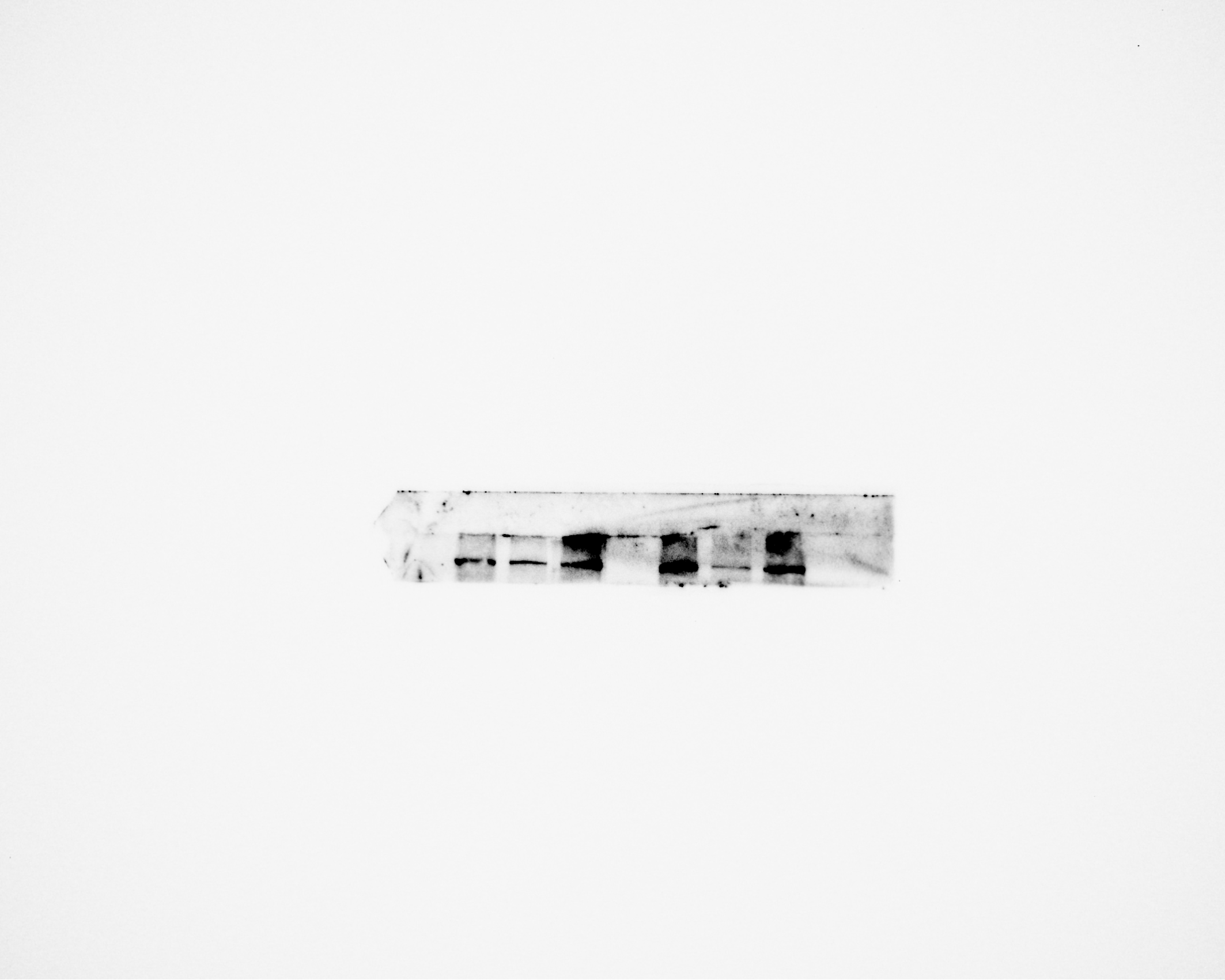

Supplement: Supplementary file 1 [file DataSheet1.zip › Original WB data/Fig 6&S1-WB/Fig6&S1-G(p-mtor).tif]

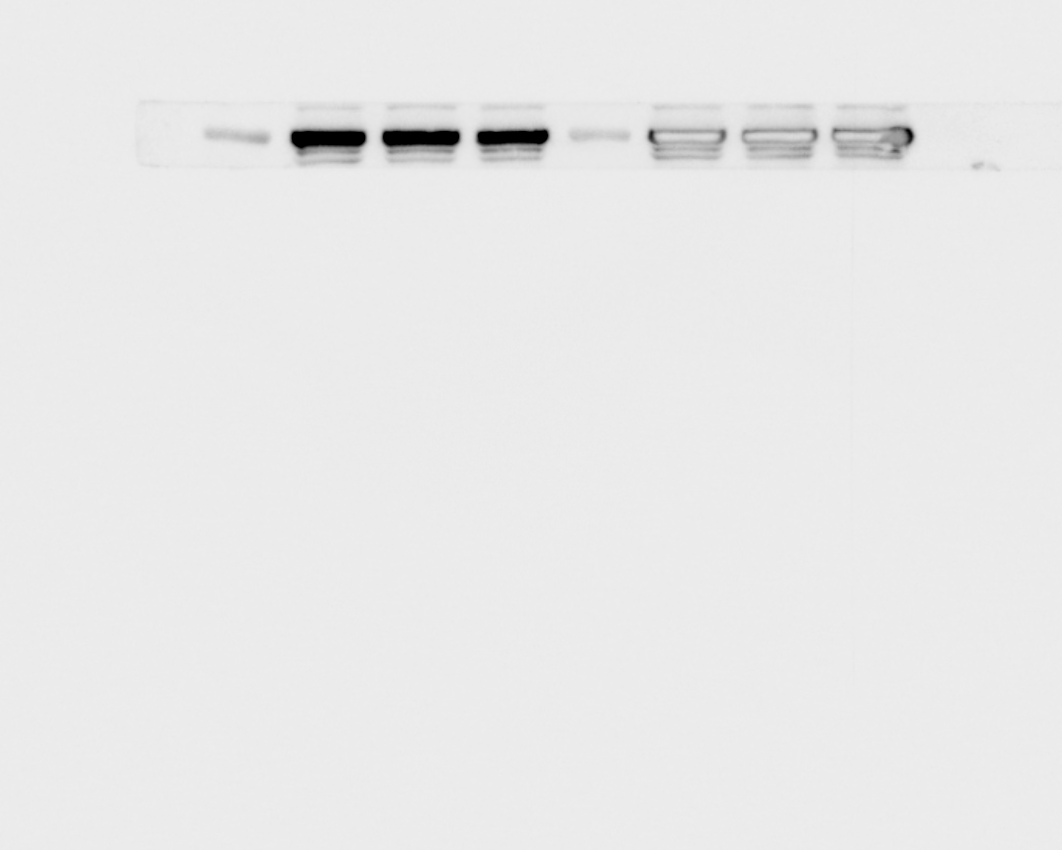

Supplement: Supplementary file 1 [file DataSheet1.zip › Original WB data/Fig 6&S1-WB/Fig6-G(gapdh).tif]

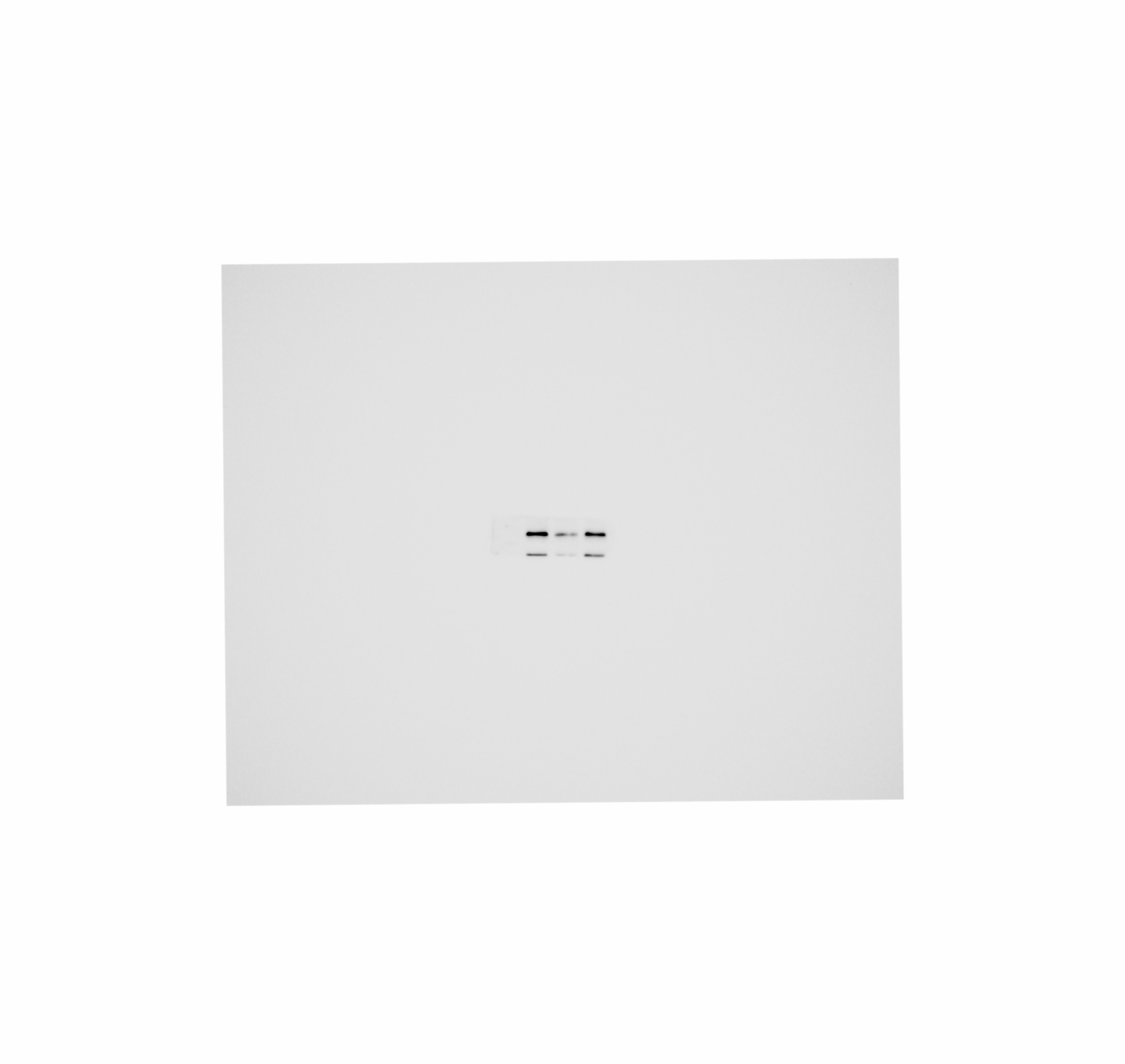

Supplement: Supplementary file 1 [file DataSheet1.zip › Original WB data/Fig 6&S1-WB/Fig6-G(p-akt).tif]

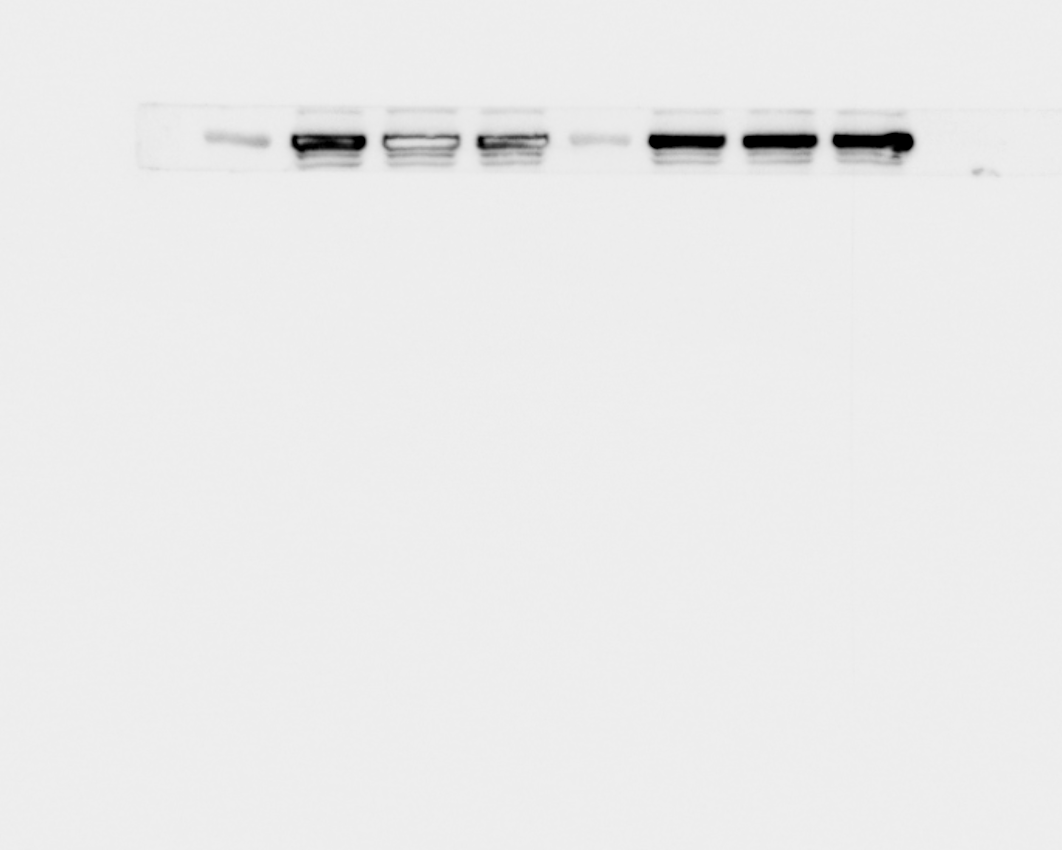

Supplement: Supplementary file 1 [file DataSheet1.zip › Original WB data/Fig 6&S1-WB/FigS1-G(gapdh).tif]

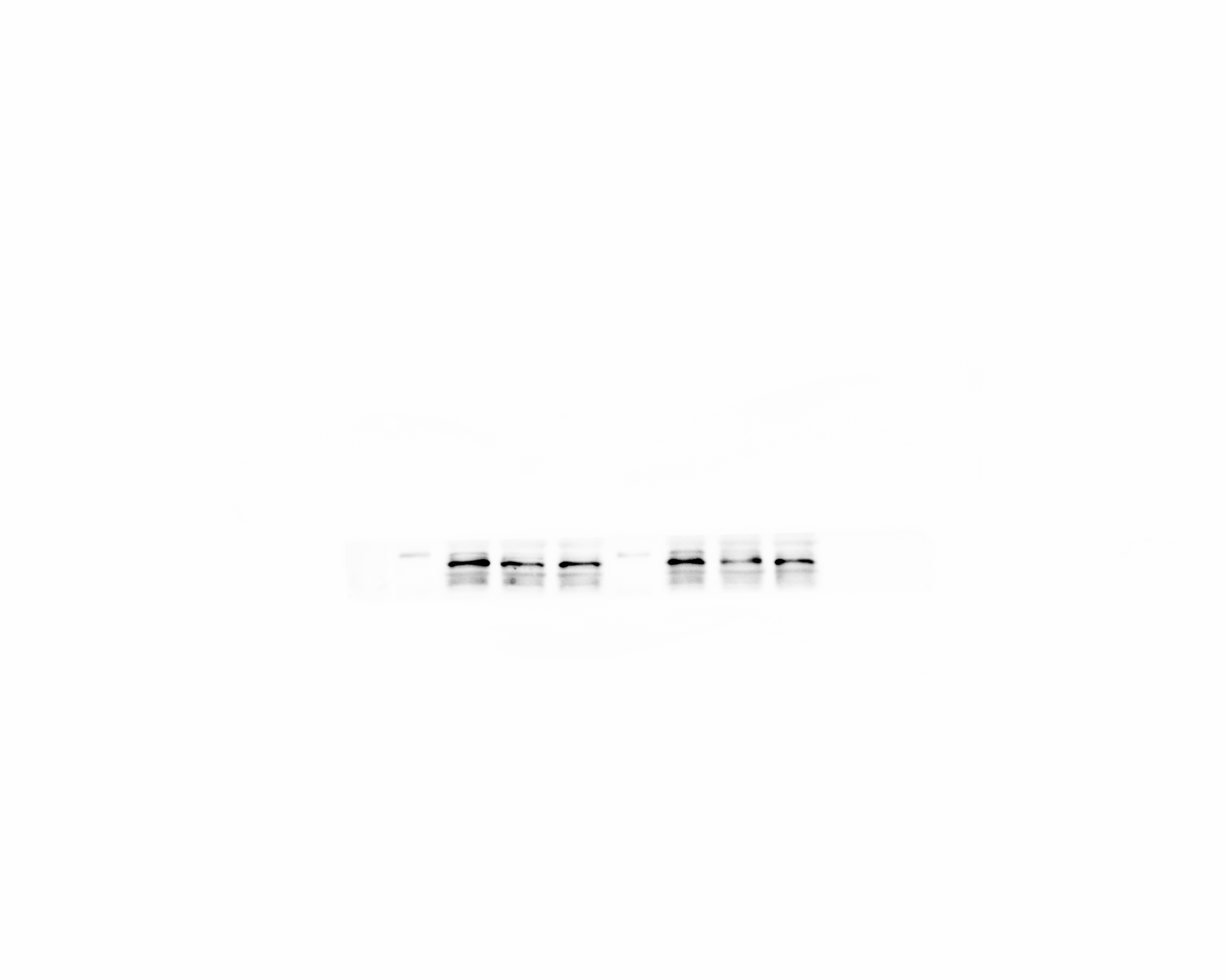

Supplement: Supplementary file 1 [file DataSheet1.zip › Original WB data/Fig 6&S1-WB/FigS1-G(p-akt).tif]

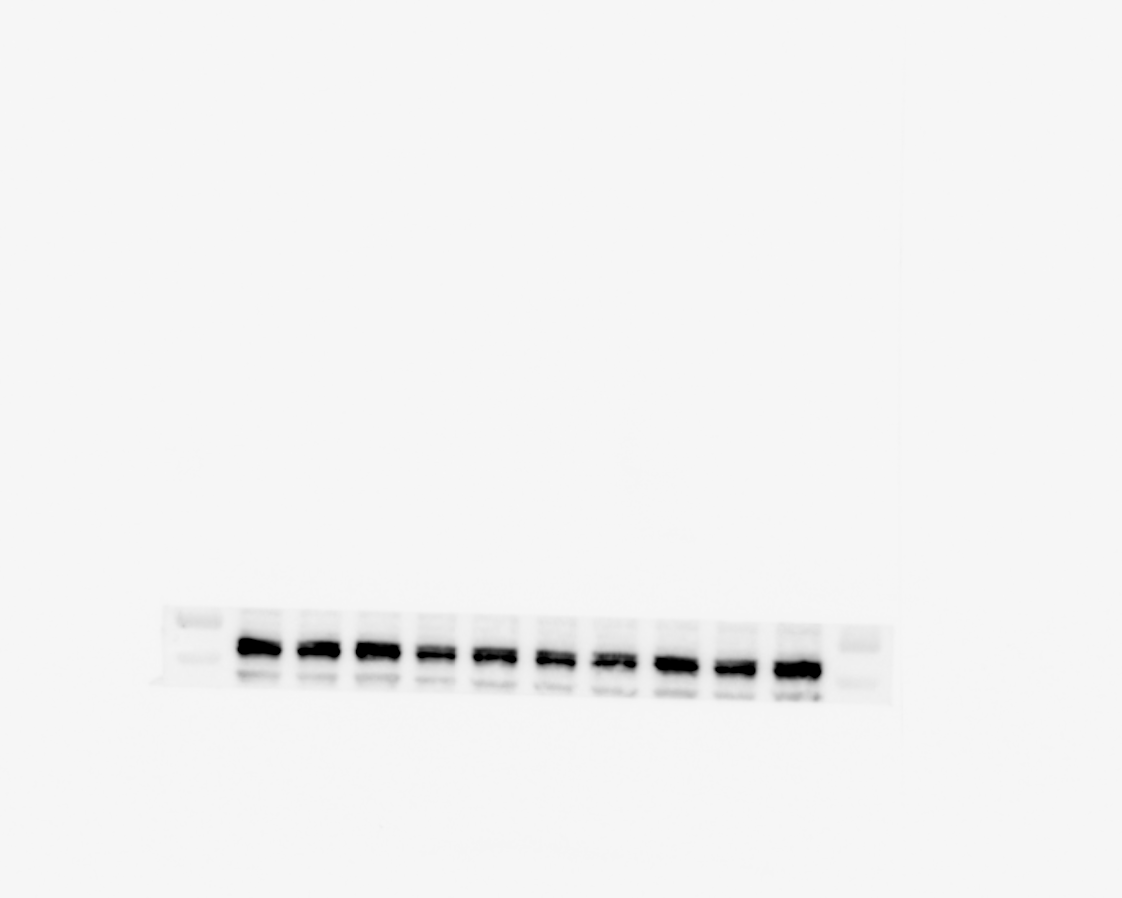

Supplement: Supplementary file 1 [file DataSheet1.zip › Original WB data/Fig 7-WB/AKT.tif]

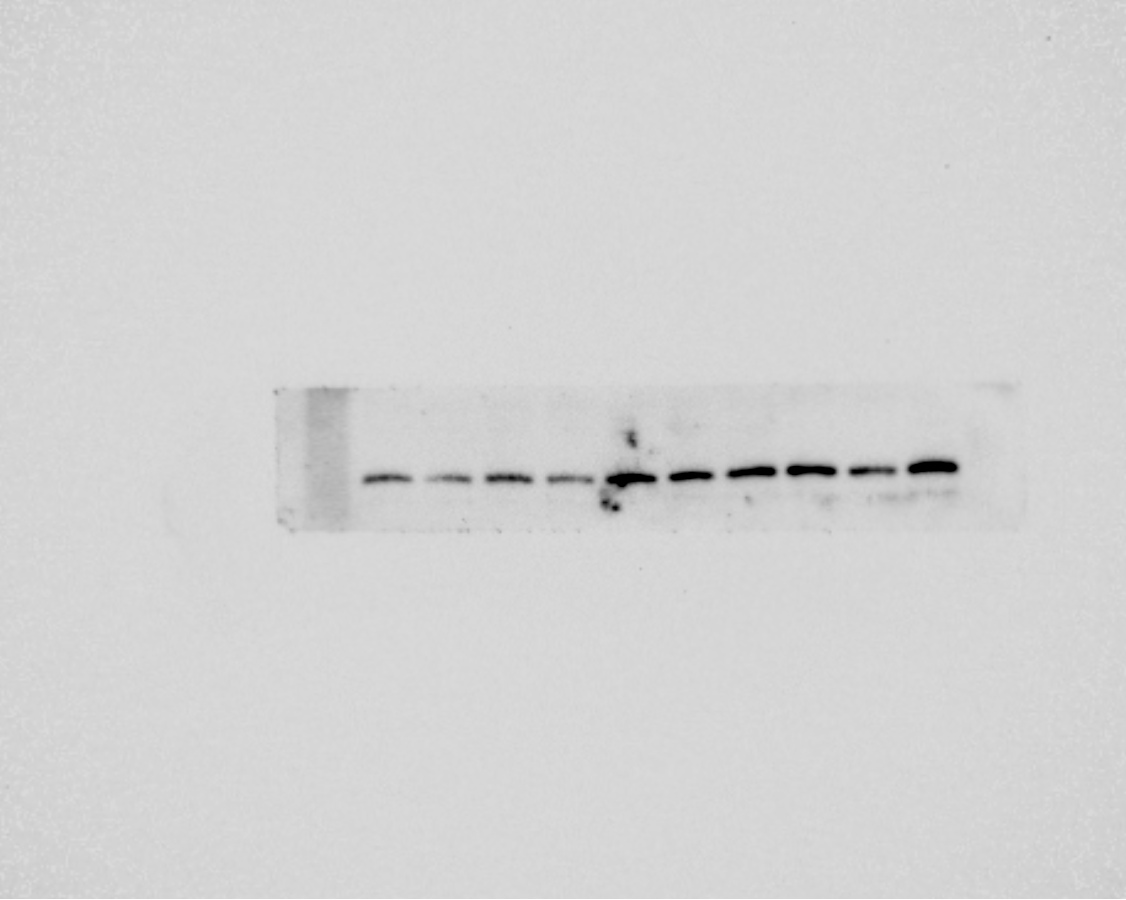

Supplement: Supplementary file 1 [file DataSheet1.zip › Original WB data/Fig 7-WB/bax.tif]

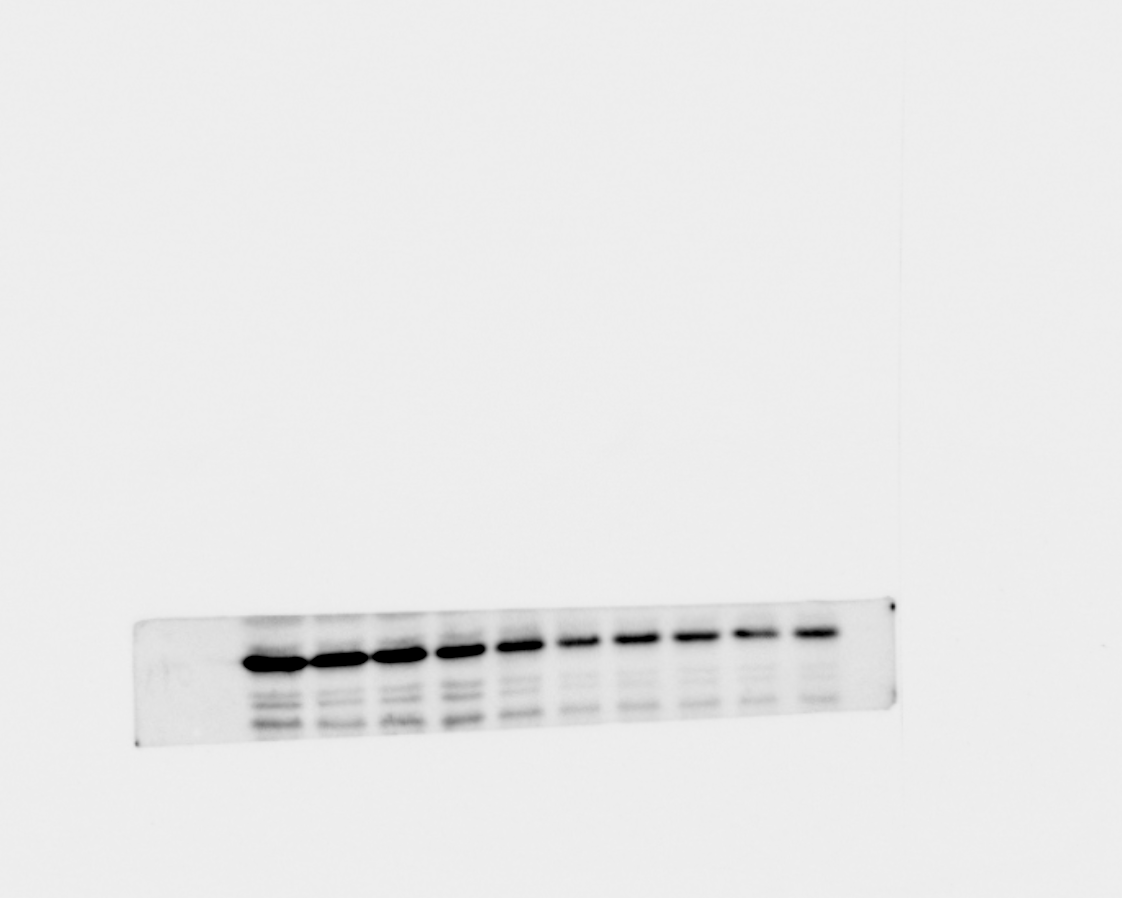

Supplement: Supplementary file 1 [file DataSheet1.zip › Original WB data/Fig 7-WB/bcl2.tif]

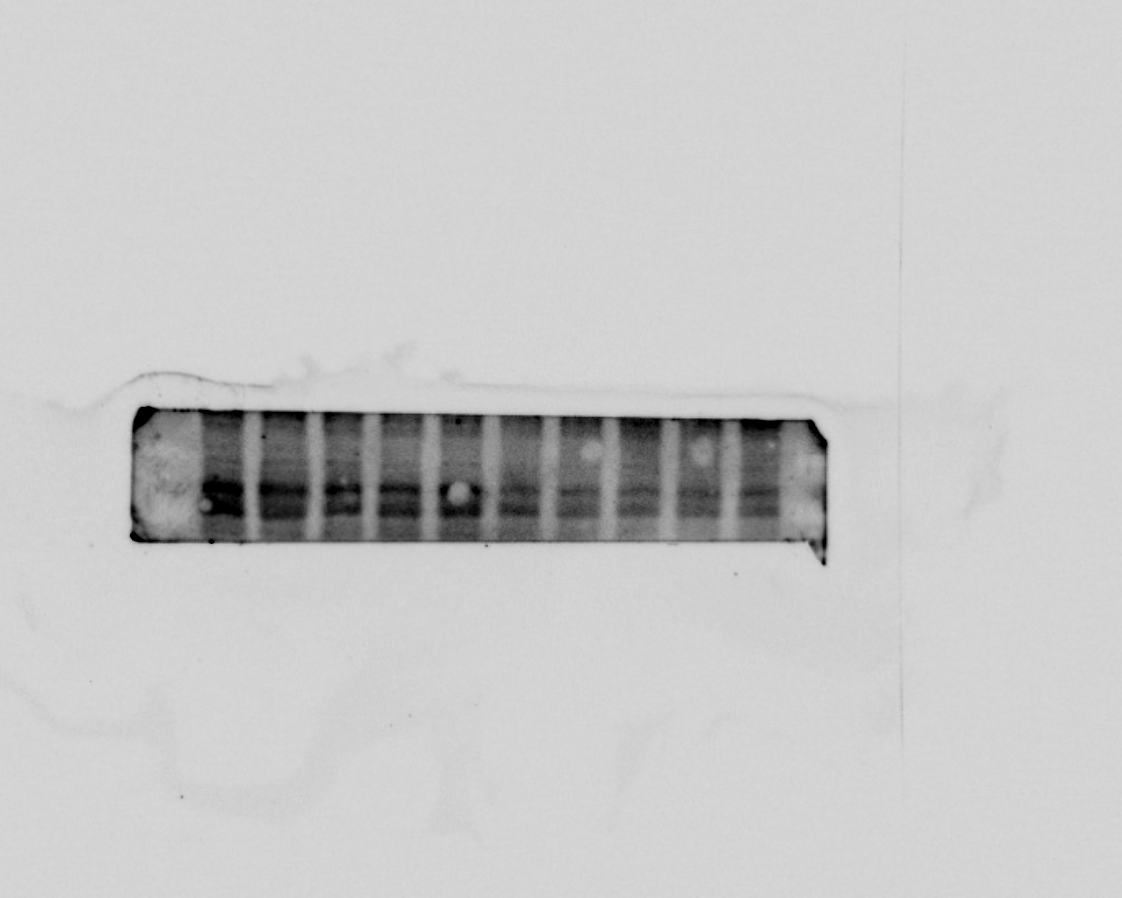

Supplement: Supplementary file 1 [file DataSheet1.zip › Original WB data/Fig 7-WB/Cyclin D1.tif]

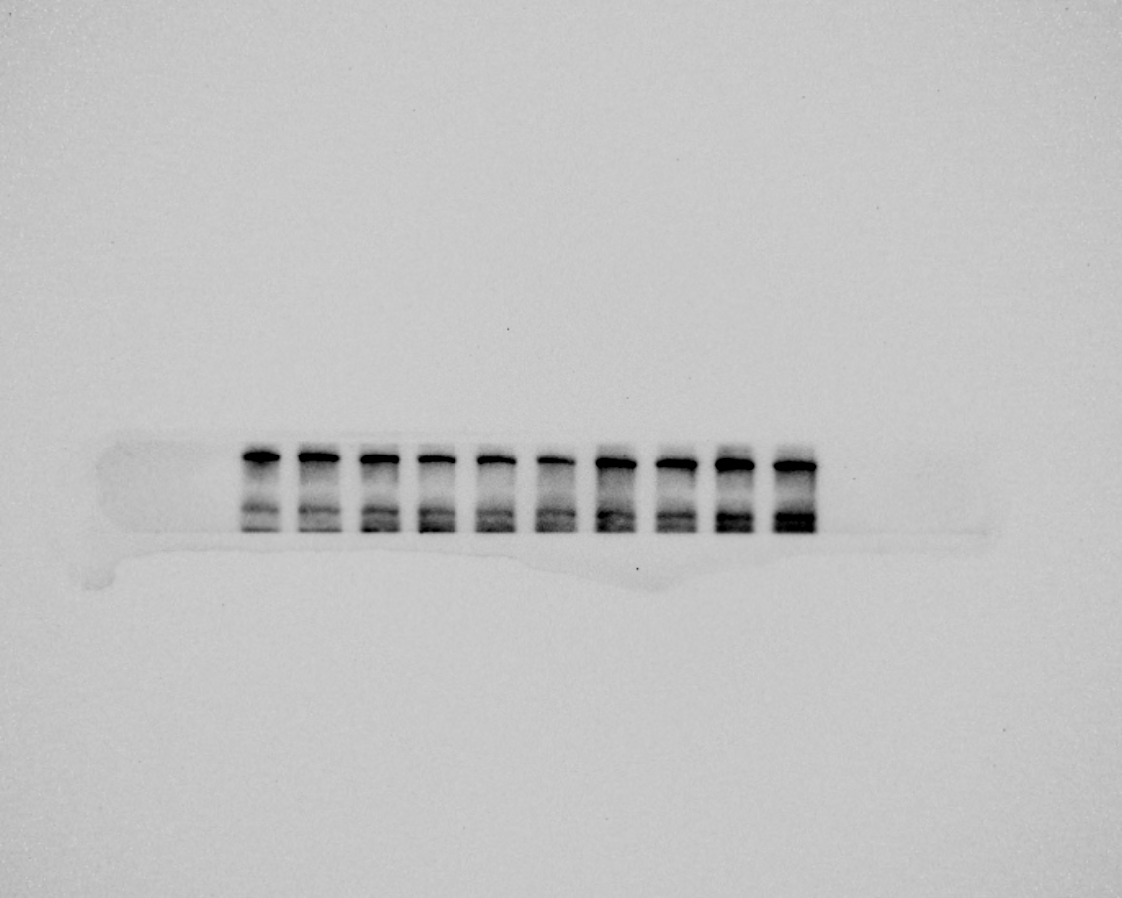

Supplement: Supplementary file 1 [file DataSheet1.zip › Original WB data/Fig 7-WB/gapdh.tif]

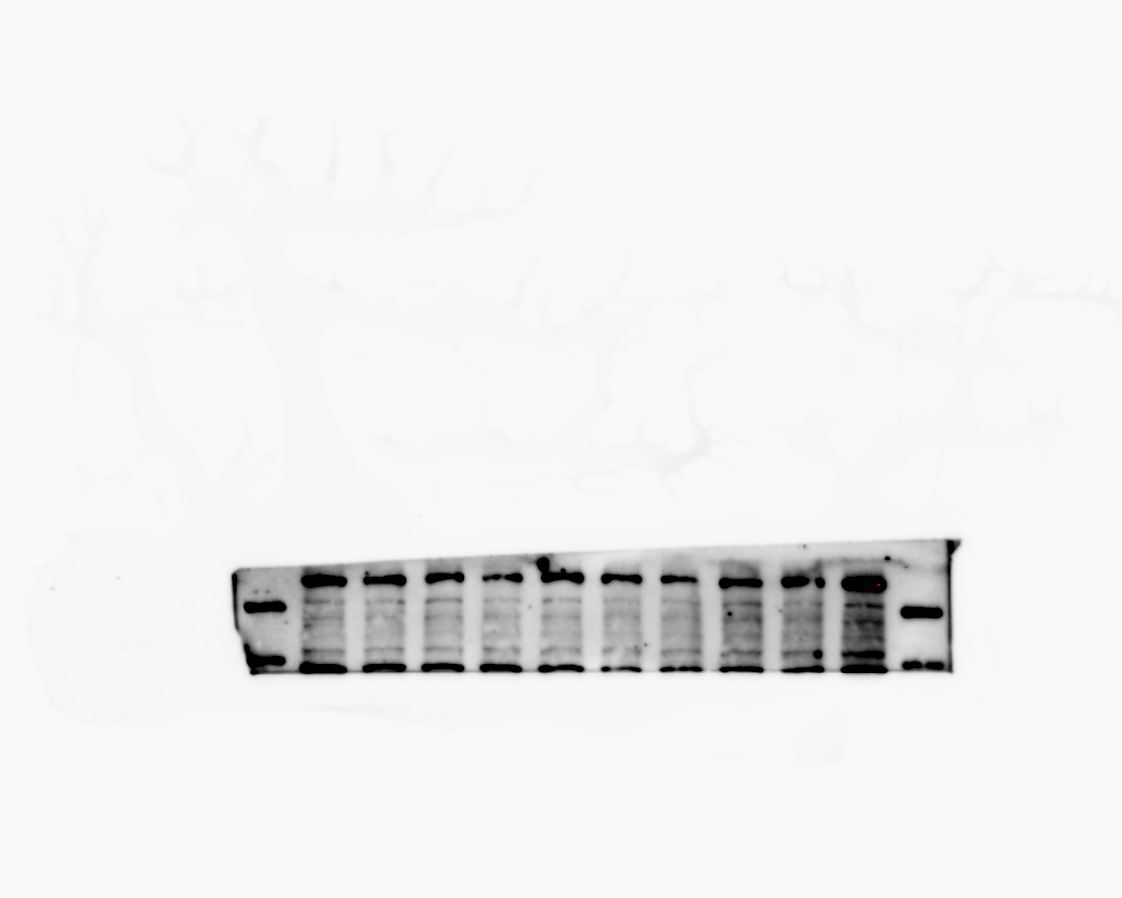

Supplement: Supplementary file 1 [file DataSheet1.zip › Original WB data/Fig 7-WB/mTOR.tif]

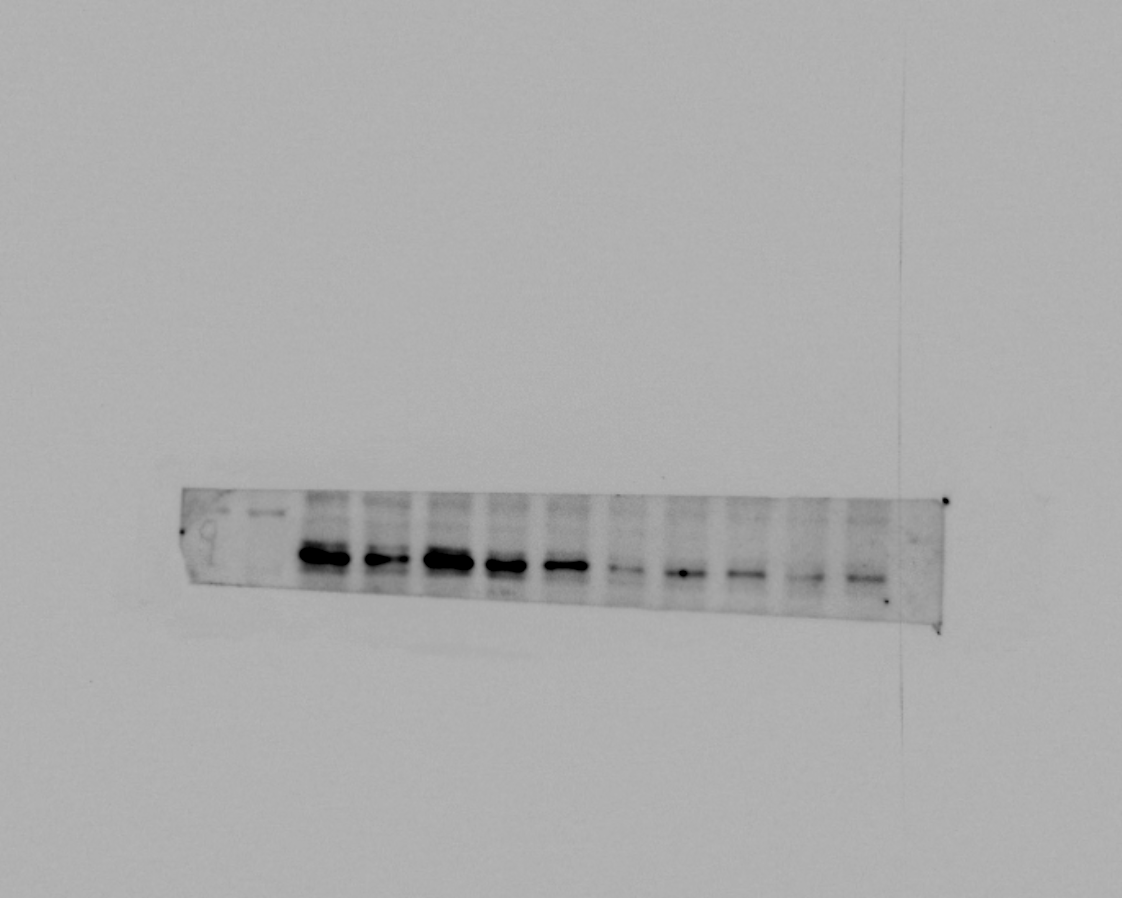

Supplement: Supplementary file 1 [file DataSheet1.zip › Original WB data/Fig 7-WB/p-AKT.tif]

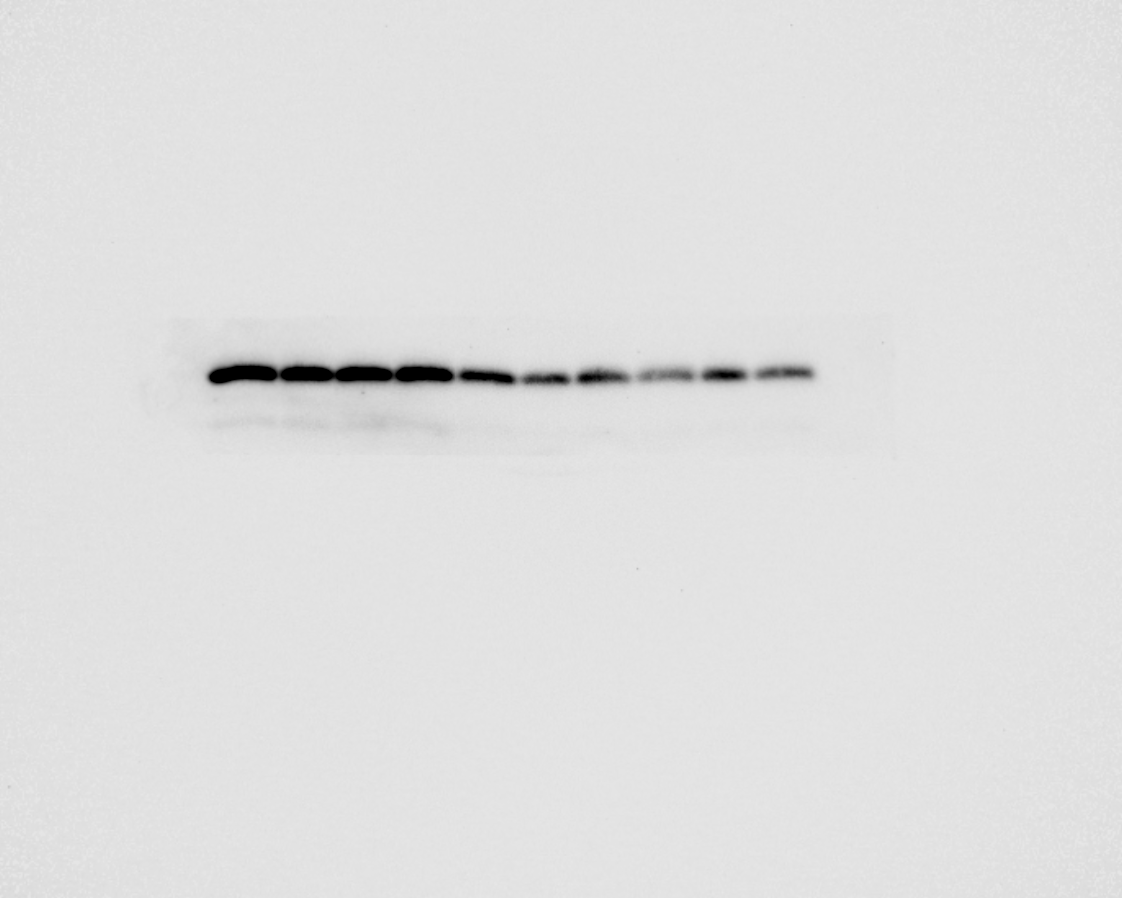

Supplement: Supplementary file 1 [file DataSheet1.zip › Original WB data/Fig 7-WB/p-mtor.tif]
